# Aberrations in the Cross-Talks Among Redox, Nuclear Factor-κB, and Wnt/β-Catenin Pathway Signaling Underpin Myalgic Encephalomyelitis and Chronic Fatigue Syndrome

**DOI:** 10.3389/fpsyt.2022.822382

**Published:** 2022-05-06

**Authors:** Michael Maes, Marta Kubera, Magdalena Kotańska

**Affiliations:** ^1^Department of Psychiatry, Faculty of Medicine, Chulalongkorn University, Bangkok, Thailand; ^2^Department of Psychiatry, Medical University of Plovdiv, Plovdiv, Bulgaria; ^3^IMPACT Strategic Research Center, Deakin University, Geelong, VIC, Australia; ^4^Laboratory of Immunoendocrinology, Department of Experimental Neuroendocrinology, Maj Institute of Pharmacology, Polish Academy of Sciences, Kraków, Poland; ^5^Department of Pharmacological Screening, Medical College, Jagiellonian University, Kraków, Poland

**Keywords:** chronic fatigue syndrome (CFS), inflammation, neuro-immune, oxidative and nitrosative stress, myalgic encephalomyelitis, antioxidants, bacterial translocation

## Abstract

There is evidence that chronic fatigue spectrum disorders (CFAS-Ds), including myalgic encephalomyelitis (ME), chronic fatigue syndrome (CFS), and chronic fatigue with physiosomatic symptoms including when due to comorbid medical disease, are characterized by neuroimmune and neuro-oxidative biomarkers. This study was performed to delineate the protein–protein interaction (PPI) network of CFAS-D and to discover the pathways, molecular patterns, and domains enriched in their PPI network. We performed network, enrichment, and annotation analyses using differentially expressed proteins and metabolics, which were established in patients with CFAS-D. The PPI network analysis revealed that the backbone of the highly connective CFAS-D network comprises *NFKB1*, *CTNNB1*, *ALB*, peroxides, *NOS2*, tumor necrosis factor (*TNF*), and interleukin-6 (*IL-6*) and that the network comprises interconnected immune-oxidative-nitrosative and Wnt/β-catenin subnetworks. Multiomics enrichment analysis shows that the CFAS-D network is highly significantly associated with cellular (antioxidant) detoxification, hydrogen peroxide metabolic process, peroxidase and oxidoreductase activity, interleukin-10 (IL-10) anti-inflammatory signaling and neurodegenerative canonical Wnt, the β-catenin complex, cadherin domains, cell–cell junctions and TLR2/4 pathways, and the transcription factors nuclear factor kappa B (NF-κB) and RELA. The top 10 DOID annotations of the CFAS-D network include four intestinal, three immune system disorders, cancer, and infectious disease. The custom Gene Ontology (GO) term annotation analysis revealed that the CFAS-D network is associated with a response to a toxic substance, lipopolysaccharides, bacterium, or virus. In conclusion, CFAS-D may be triggered by a variety of stimuli and their effects are mediated by aberrations in the cross-talks between redox, NF-κB, and Wnt/β-catenin signaling pathways leading to dysfunctions in multicellular organismal homeostatic processes.

## Introduction

Contemporary study of chronic fatigue spectrum disorders (CFAS-Ds), including myalgic encephalomyelitis (ME), chronic fatigue syndrome (CFS), and chronic fatigue (CF) and associated and associated physiosomatic (psychosomatic) symptoms, is plagued by a cacophony of controversies. These different approaches, sometimes even competing, comprise folk psychology (the culprit of CFAS-D is a psychological problem) and the medical approach (the culprit is one specific virus or different viruses or bacteria). The dominant view, especially in Europe, is that of the cognitive-behavioral and the biopsychosocial schools (1). This view entails that CFAS-Ds, even when due to medical disease (e.g., cancer), are the consequence of psychosocial and biological factors and negative cognitions (1–4). The Wessely model, for example, conceptualizes that the effects of a trigger factor, which may be a virus, are mediated by boom and bust activity and bed rest and the Vercoulen model considers that CFAS-D symptoms are aggravated by causal attributions and reduced physical activity (1). Nevertheless, it appears that the label “biopsychosocial” is more window dressing than the actual approach because, in fact, folk psychology statements abound in their publications, as, for example, “it is in the mind,” “they think themselves ill,” and “it is a disorder of perception, whereby patients think those symptoms are the consequence of a virus” [review: 2]. Nevertheless, this folk psychology approach is embraced by the National Health Service (NHS), the Lancet, and national healthcare system all over Europe (e.g., United Kingdom, France, Sweden, Benelux).

It is incomprehensible that expert committees make consensus diagnostic criteria of ME/CFS (including the 1994 International Research Case Definitions and the 2003 Canadian Consensus Criteria), whereas, in fact, these new diagnostic classes were never validated, for example, by using machine learning techniques and there is no evidence that the case definitions have reliability and replicability validity (2, 4, 5). One diagnostic criterion of both the abovementioned case definitions is that ME and CFS are not secondarily to medical disorders (6). However, even CFS and ME, which are not secondary to any medical disorder, show a high comorbidity with symptoms of irritable bowel syndrome and depression (7), while CFAS-D symptoms are hallmarks of autoimmune, infectious, immune system, and psychiatric disorders, including major depression and schizophrenia and many more medical conditions (1, 3, 7–10). CFAS-D symptoms are reflective manifestation of the symptomatome of, for example, coronavirus disease 2019 (COVID-19), chronic kidney disease with hemodialysis, schizophrenia, and rheumatoid arthritis (9–14), suggesting that CFAS-D symptoms are part of the core of these medical disorders and not the result of some “it is in the mind” process, if there would exist such a process.

It was not until 2010–2013, when Maes et al. (1, 3, 15) launched a new biomedical model that more targeted medical research into CFAS-D began. These biomedical models of Maes et al. conceptualized CFAS-D as medical conditions whereby trigger factors such as immune activation, bacterial and viral infections, and a reduced protectome due to, for example, reduced antioxidant levels may cause multiple adverse outcome pathways (AOPs) leading to the phenome of CFAS-D. The latter comprises CF symptoms, neurocognitive impairments, and depressive, sleep, gastrointestinal, and autonomic symptoms, hyperesthesia, and fibromyalgia-like symptoms, a flu-like malaise including post-exertional malaise (1, 3, 15). Furthermore, we conceptualized physiosomatic symptoms accompanying CFAS-D as being the consequence of different AOPs and, therefore, relabeled those symptoms as “physiosomatic” symptoms (16, 17).

The key AOPs of CFAS-D (including those that are secondary to medical disorders such as COVID-19, chronic kidney disease, rheumatoid arthritis, schizophrenia, and major depression, which we established in these studies between 2001 and 2021) are shown in Table 1. In addition, we also discovered that ME/CFS is characterized by increased immunoglobulin A/immunoglobulin M (IgA/IgM) responses to Gram-negative bacteria indicating increased bacterial or lipopolysaccharide (LPS) translocation and IgM-mediated autoimmune responses to a number of neoantigens including malondialdehyde (MDA), azelaic acid, and nitrosylated proteins, indicating increased nitrosylation (18, 19).

**TABLE 1:** The key adverse outcome pathways (AOPs) of the chronic fatigue spectrum disorders (CFAS-Ds), including those that are secondary to medical disorders such as coronavirus disease 2019 (COVID-19), chronic kidney disease, rheumatoid arthritis, schizophrenia, and major depression.

Pathways	Biomarkers
Oxidative and nitrosative stress (O&NS)	Increased hydroperoxides (H_2_O_2_) and inducible nitric oxide (NOS) synthase Reduced antioxidant balances, including coenzyme Q10, zinc, dehydroepiandrosterone (DHEA), glutathione peroxidase (GPX)1, and albumin (ALB)
A multitude of immune disorders	Changes in CD (cluster of differentiation) markers on peripheral blood mononuclear cells, including CD3, CD8, CD19, CD38, CD69, and HLA-DR
	Increased levels of interleukin (IL)-1α and IL-1β, IL-6, IL-10, tumor necrosis factor (TNF)-α and TNF receptors (TNFRs), soluble IL-1R antagonist (sIL-1RA), granulocyte-macrophage colony-stimulating factor (CSF2), high mobility group box protein 1 HMGB1), neopterin, C-reactive protein (CRP), haptoglobin (HP), C–C motif chemokine ligand (CCL)2, CCL4, CCL11, cyclo-oxygenase-2 (PTGS2), soluble Toll Like Receptor (TLR)4, lysozyme (LYZ), and elastase (ELANE)
Increased levels transcriptional factors	Increased production of nuclear-factor (NF)-κB
Changes in cell-cell-junction and Wnt pathway proteins	Changes in catenin-β (CTNNB1), claudin-5 (CLDN5), occludin (OCLN), Dickkopf Wnt Signaling Pathway Inhibitor 1 (DKK1), and R-spondin-1 (RSPO1)
Altered activity of the endogenous opioid system	Altered levels of OPRK1 (κ opioid receptor) and OPRM1 (Mu opioid receptor)
Induction of the tryptophan catabolite (TRYCAT) pathway	Increased levels of some neurotoxic TRYCATs, including 3-OH-kynurenine (3HK)

All in all, these findings show that multiple differentially expressed proteins (DEPs) and metabolic pathways are involved in the pathophysiology of CFAS-D. However, no study has delineated the protein–protein interaction (PPI) and metabolic-protein interactions (MPIs) networks of CFAS-D and the biological processes, molecular functions and complexes, cellular components, pathways, transcriptional regulatory relationships, protein domains, and human disease annotations, which are associated with the PPI and MPI networks of CFAS-D.

Hence, we have conducted network, enrichment, and annotation analyses to delineate the hotspots in the CFAS-D network and the top functions and paths enriched in the networks. This is important because the most influential genes, metabolics, and pathways may constitute new drug targets to treat CFAS-D. Moreover, such analyses may disclose the putative trigger factors of the CFAS-D interactome and its associations with comorbid medical disorders. As such, these enrichment and annotation analyses may help to explain the strong comorbidity of CFAS-D with immune, infectious, and neuropsychiatric disorders and the possible shared pathophysiological core, which may underpin CFAS-D.

## Materials and Methods

### Selection of Seed Proteins and Metabolic Markers

“This study is a secondary data analysis on existing data using open, deidentified, and non-coded data sets and, therefore, this is non-human subject study, which is not subjected to Institutional Review Board approval” (20). In case–control studies, we have previously identified the metabolic pathways and differentially expressed proteins (DEPs) in CFAS-D, including when due to comorbid medical disease. Almost all the biomarkers included in this study were extracted from our studies on ME/CFS and CF-like symptoms with a duration > 6 months in comorbid disorders, including major chronic kidney disease with hemodialysis, depression, schizophrenia, and rheumatoid arthritis [Electronic Supplementary File (ESF), 1, References]. One study was performed on patients with CF-like symptoms due to acute COVID-19 infection. We were able to include: (a) 42 DEPs, namely, angiotensin-converting enzyme 2 (ACE2), advanced glycosylation end-product specific receptor (AGER), agrin (AGRN), ALB, CCL2, CCL4, CCL11, CD3D, CD8A, CD19, CD38, CD69, HLA-DR, creatine phosphokinase (CKM), CLDN5, CSF2, CRP, CTNNB1, DKK1, ELANE, GPX1, hemoglobin A1 (HbA1), HP, HMGB1, interleukin-1α (IL-1α), interleukin-1β (IL-1β), IL-1RN (IL-1RA), IL-6, IL-10, LYZ, NFKB1 (NF-κB), NOS2, OCLN, opioid receptor Kappa 1 (OPRK1), opioid receptor Mu 1 (OPRM1), proopiomelanocortin (POMC) as precursor of β-endorphins, PTGS2, RSPO1, TLR4, TNF (TNF-α), TNFRSF1A (TNFR60), and TNFRSF1B (TNFR80) and (b) 12 metabolic Kyoto Encyclopedia of Genes and Genomes (KEGG)^1^ pathways, namely, C01290 (lactosylceramide), C02470 (xanthurenate), C10164 (picolinic acid), C03227 [3-OH-L-kynurenine (3OHK)], C11378 (coenzyme Q10), C00038 (zinc), C00070 (copper), C06428 [eicosapentaenoic acid (EPA)], C00027 [hydrogen peroxides (H_2_O_2_)], C19440 [malondialdehyde (MDA)], C004555 (DHEA), and C05926 (neopterin).

### Protein–Protein Interaction Network Construction and Enrichment and Annotation Analyses

The network, enrichment, and annotation analyses were conducted, as reviewed previously (20). In brief, we constructed two network subtypes, the first was constructed using the abovementioned DEPs whereby the physical interactions between the DEPs were visualized using STRING version 11.0^2^ (minimum required interaction score: 0.400; active interaction sources: experiments, textmining, and databases; and set organism: *Homo sapiens*) and Cytoscape^3^. The second was constructed using OmicsNet^4^ using the abovementioned metabolites and examining the MPI based on the KEGG reactions. Consequently, the genes interacting with the metabolics in the different subnetworks were used in STRING to build a giant network based on our seed genes enlarged with the MPI-derived DEPs, which were, consequently, analyzed in OmicsNet to construct a composite network consisting of metabolites and DEPs first entering MPIs and then the PPIs (analyzed with IntAct molecular interaction database)^5^ with a targeted incorporation of TF protein interactions (TFPIs) (analyzed using TTRUST)^6^.

Network features were calculated using STRING and the Cytoscape plugin NetworkAnalyzer and comprise number of nodes, number of edges and expected number of edges, average node degree, network diameter and radius, characteristic path length, and network density and heterogeneity. The top hubs (high degree) and bottlenecks (high betweenness centrality) were calculated and used to delineate the backbone of the network, i.e., the top 7 hubs and the top 2 non-hub bottlenecks. We used Markov Clustering (MCL) employing STRING to discover communalities of interconnected nodes, which display similar attributes and/or functions.

We examined the different networks and MCL subnetworks for their enrichment scores and annotated terms and these analyses were also performed on the downregulate seed genes and the hotspots of the enlarged network. Enrichment/annotation analyses were performed using STRING, Enrichr^7^, OmicsNet, MetaScape^8^, inBio Discover^9^, and the R package ClusterProfiler. Heatmaps were produced using Appyter and MetaScape. Functional enrichments were established using the Gene Ontology (GO) biological processes, the GO molecular functions and the GO cellular components, STRING local network clusters, the KEGG pathways, Reactome pathways (the European Bioinformatics Institute Pathway Database)^10^, PANTHER biological processes (PANTHER---Gene List Analysis)^11^, TTRUST transcriptional regulatory relationships, InterPro domains (InterPro)^12^, Wikipathways (WikiPathways - WikiPathways), and DOID annotations of human diseases (Disease Ontology---Institute for Genome Sciences, University of Maryland)^13^. We also performed Molecular Complex Detection (MCODE) (using Metascape) to delineate small molecular complexes. In this study, all the results are shown as *p* (or *q*) values corrected for false discovery rate (FDR).

## Results

### Differentially Expressed Protein Protein–Protein Interaction Network Topography of Chronic Fatigue Syndrome

**FIGURE 1:**
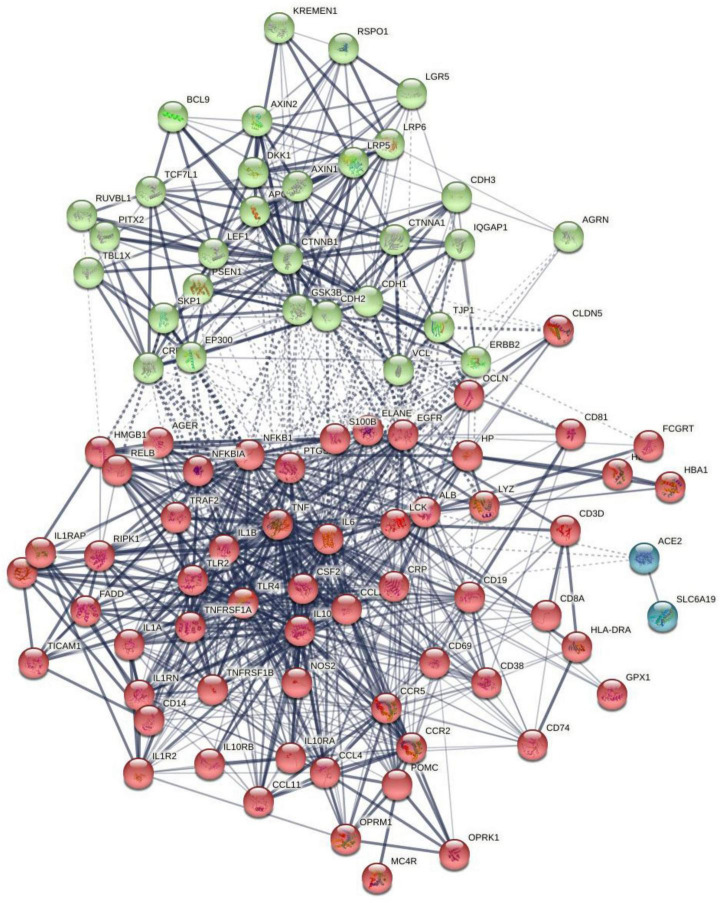
The first-order protein–protein interaction network of chronic fatigue spectrum disorders. Markov Clustering (MCL) cluster analysis found two subnetworks: (1) a first immune subnetwork (red color) was centered around NFKB1, tumor necrosis factor (TNF), interleukin-6 (IL-6), interleukin-10 (IL-10), interleukin-1 (IL-1), TLR4, etc., and (2) a second Wnt/β-catenin subnetwork (green nodes) centered around CTNNB1. The solid and dotted lines represent connections inside and between clusters, respectively. Different colors represent the various clusters.

Figure 1 shows the first-order protein network of CFAS-D. This network comprises 91 nodes and 938 edges, exceeding the expected number of edges (*n* = 323) with a *p*-enrichment value of 1.0E-16. The network has an average local clustering coefficient of 0.683 and average node degree of 20.6, with a network diameter of 4, radius of 2, a characteristic path length = 1.978, network density = 0.229, and heterogeneity = 0.682. The top 7 hubs (highest degree) were in descending order of importance: TNF (degree = 63), IL-6 (61), IL-1β (58), ALB (54), TLR4 (47), IL-10 (47), and CTNNB1 (46). The top two non-hub bottlenecks were: EGFR (0.066) and TLR2 (0.0188). EGFR and TLR were the top 5 and 9 in the bottleneck list (top 3 in descending order was ALB, CTNNB1, and TNF). The network of the seed proteins showed 42 nodes and 311 edges, thus, exceeding the expected number of edges (*n* = 70) with a *p*-enrichment value of 1.0E-16. The network has an average local clustering coefficient of 0.74 and an average node degree of 14.8.

Markov Clustering analysis (inflation parameter of 2) resulted in two protein subnetworks, as shown in Figure 1. The first subnetwork contains predominantly immune genes, such as EGFR, ALB, AGER, HP, GPX1, CD19, etc., and the second subnetwork contains Wnt and cell–cell junction associated genes, including CTNNB1, AGRN, RSPO1, and DKK1. The major connectors (switches) of both the subnetworks were CTNNB1 (belongs to subnetwork 2) and ALB and IL-6 (nodes in subnetwork 1). CTNNB1 showed relevant interactions (confidence level > 0.4) with DKK1 and RSPO1 and with 11 subnetwork 1 seed genes (e.g., NFKB1, ALB, OCLN, TLR4, HMGB1). ALB showed significant interactions with 25 other subnetwork 1 genes and with CTNNB1, AGRN, and DKK1. IL-6 showed interactions with CTNNB1, DKK1, and AGRN and 28 other subnetwork 1 seed nodes. In the first-order non-seed genes, we found that EGFR was connected with 3 subnetwork 2 seed genes (GRN, CTNNB1, and DKK1) and with 19 seed genes in subnetwork 1.

### Multiomics Analysis Including the Oxidative Stress-Associated Metabolites

**FIGURE 2:**
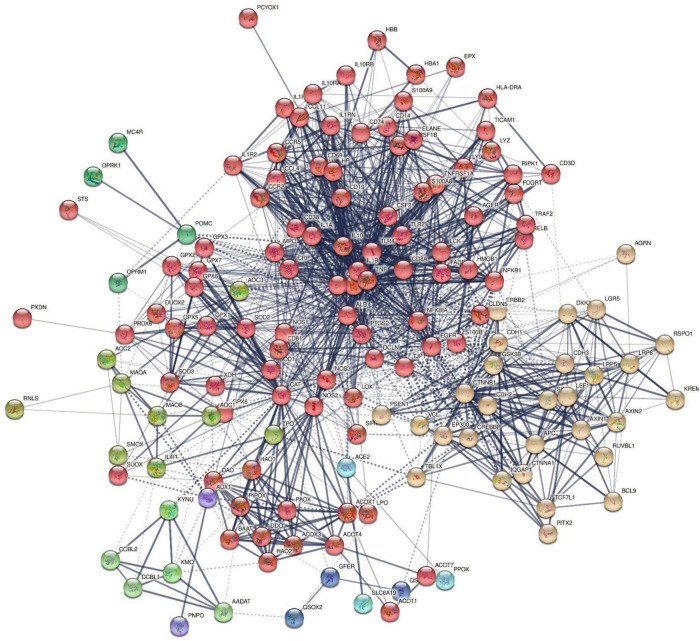
First-order protein network of chronic fatigue spectrum disorders. MCL cluster analysis found two major subnetworks: (1) a first immune, oxidative, and nitrosative subnetwork (IO&NS) (red color) was centered around NOS2, NFKB1, IL-10, etc, and (2) a second Wnt/β-catenin subnetwork (yellow nodes) was centered around CTNNB1. The tryptophan catabolite pathway [kynurenine hydroxylase (KYNU) and kynurenine 3-monooxygenase (KMO)] and opioid [opioid receptor Kappa 1 (OPRK1) and opioid receptor Mu 1 (OPRM1)] genes appear to be spin-offs of the IO&NS subnetworks. The solid and dotted lines represent connections inside and between clusters, respectively. Different colors represent the various clusters.

In order to construct a second giant network including proteins interconnecting with the metabolites, we entered the latter in OmicsNet analysis and examined the MPIs (using the KEGG and IntAct and using only the first-order MPIs). We found 5 subnetworks: one centered around hydroperoxides (46 nodes), another centered around EPA (6 nodes), 6 centered around 3OHK, 4 centered around nitric oxide (NO), and 3 centered around DHEA (albeit some overlapping). The 59 nodes coupled with the nodes from the first network (see Figure 2) were consequently examined using STRING and NetworkAnalyzer. Figure 2 shows the network based on this combination of DEPs. This network comprises 147 nodes and 1,407 edges, exceeding the expected number of edges (*n* = 413) with a *p*-enrichment value of 1.0E-16. The network has an average local clustering coefficient of 0.624 and average node degree of 19.1, with a network diameter of 5, radius of 3, characteristic path length = 2.278, network density = 0.131, and heterogeneity = 0.799. The top 7 hubs (highest degree) were in descending order of importance: TNF (degree = 82), IL-6 (79), ALB (75), IL-1β (74), catalase (CAT) (59), IL-10 (57), and TLR4 (57). The top 5 bottlenecks were CAT (0.1534), ALB (0.1212), CTNNB1 (0.0918), TNF (0.08867), and IL-6 (0.06755). The first non-hub bottlenecks were EGFR (0.04909) and NOS2 (0.04572).

Figure 2 shows the results of MCL cluster analysis (inflation parameter of 1.7) displaying two significant protein subnetworks, a first comprising immune and nitro-oxidative stress genes [see Figure 1; this cluster is now renamed the immune-inflammatory, oxidative, and nitrosative (IO&NS) cluster] and a second Wnt/β-catenin cluster. There were also some communities with only few nodes, for example, one centered around kynurenic acid (KYNA) and kynurenine 3-monooxygenase (KMO).

**FIGURE 3:**
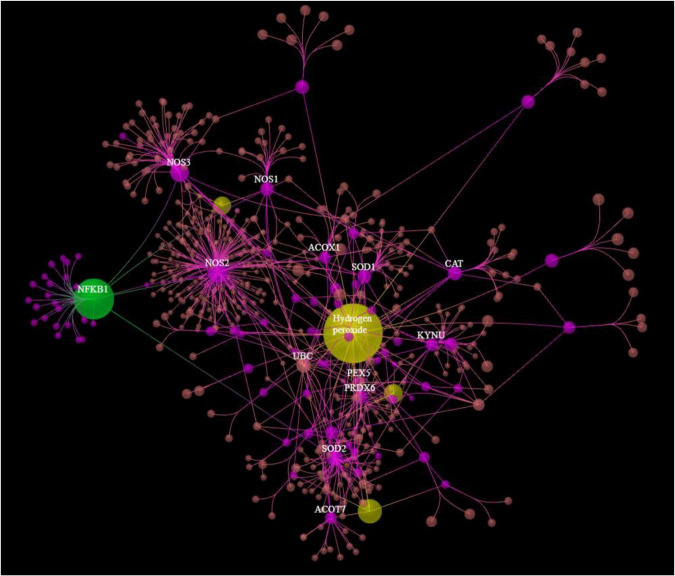
Results of OmicsNet analysis, which included all the genes of the network with integration of the metabolics and NFKB1. Metabolics are shown in yellow color and NFKB1 is shown in green color. 3HK: 3-hydroxykynurenine; NO: nitric oxide; EPA: eicosapentaenoic acid.

Figure 3 shows the results of OmicsNet analysis, which included all the *IO*&*NS/Wnt* genes of the network given in Figure 2 with integration of the metabolics and NFKB (as transcriptional factor). This network was constructed using two interaction types, namely, MPI (first rank) and PPI (second rank) and included 574 nodes and 694 edges. The top hubs (including non-seeds) in this network were NOS2 (177), NOS3 (63), SOD2 (57), PRDX6 (47), peroxides (45), SOD1 (41), KYNU (32), and NFKB1 (29). We also examined the hubs of the network built with the PPIs entered in first order and MPI in second order (2,593 nodes and 4,063 edges) and found that the top 3 hubs were in descending order of importance: NFKB1 (307), CTNNB1 (153), and ALB (152). As such, the common backbone of the different networks (top 3 of each network) in this study consists of NFKB1, CTNNB1, ALB, peroxides, NOS2, TNF, and IL-6.

Table 2 shows the results of PANTHER functional explorer analyses. The top PANTHER molecular functions, which were enriched in this network, revolved around protein binding, peroxidase, and oxidoreductase activity and the top PANTHER cellular components were the cytosol, protein-containing complex, cytoplasm, and the nucleus.

**TABLE 2:** PANTHER molecular function and components terms associated with chronic fatigue spectrum disorders.

PANTHER molecular functions	total	expected	hits	P	pFDR
Protein binding	9580	334	461	9.74E-33	1.89E-30
Peroxidase activity	31	1.08	21	5.38E-24	5.22E-22
Oxidoreductase activity	590	20.6	75	7.24E-23	4.68E-21
RNA binding	1510	52.6	107	4.94E-13	2.39E-11
Nuclear receptor activity	45	1.57	14	2.04E-10	7.93E-09
Signaling receptor binding	413	14.4	38	5.05E-08	1.63E-06
Double-stranded DNA binding	128	4.46	18	4.71E-07	1.30E-05
Antioxidant activity	26	0.906	8	1.86E-06	4.50E-05
**PANTHER cellular components**	**total**	**expected**	**hits**	**P**	**pFDR**
Cytosol	5030	157	330	1.11E-53	5.88E-52
Protein-containing complex	656	20.6	85	1.34E-29	3.54E-28
Cytoplasm	6540	205	334	3.10E-29	5.48E-28
Nucleus	6480	203	309	2.33E-20	3.08E-19
Mitochondrion	1510	47.2	94	6.79E-11	7.19E-10
Peroxisome	119	3.73	21	1.19E-10	1.05E-09
Protein-DNA complex	44	1.38	12	6.72E-09	5.09E-08

*FDR: false discovery rate.*

### Enrichment and Annotation Analyses in All the Immune-Inflammatory, Oxidative, and Nitrosative/Wnt Genes of Chronic Fatigue Spectrum Disorders

Table 3 displays the results of MCODE analysis using the KEGG, WikiPaths, the GO biological and molecular, Reactome, and PANTHER performed on the *IO*&*NS/Wnt* genes. We found five significant molecular complexes, the first represents cytokine/IL-10 signaling and a response to LPS; the second comprises cellular oxidant detoxification and response to a toxic substance; the third comprises amine oxidase reactions, hydrogen peroxide metabolic process, and degradation of β-catenin; the fourth comprises peroxisomal protein import and a carboxylic acid catabolic response; and the fifth was the same as MCODE2, as given in Table 3.

**TABLE 3:** Results of molecular complex detection (MCODE) analysis performed on the differently expressed proteins (DEPs) of chronic fatigue spectrum disorders.

MCODE Components	GO ID	Biological term	Log10 (p)
All DEPs, MCODE_ALL	GO:0098869	Cellular oxidant detoxification	−33.0
	GO:1990748	Cellular detoxification	−31.5
	GO:0097237	Cellular response to toxic substance	−30.8
All DEPs, MCODE1	R-HSA-449147	Signaling by Interleukins	−31.9
	R-HSA-6783783	Interleukin-10 signaling	−30.0
	GO:0032496	Response to lipopolysaccharide	−29.7
All DEPs, MCODE2		Same as MCODE_ALL	
All DEPs, MCODE3	R-HSA-140179	Amine Oxidase reactions	−9.0
	GO:0042743	hydrogen peroxide metabolic process	−7.2
	M31	PID BETA CATENIN DEG PATHWAY	−6.7
All DEPs, MCODE4	R-HSA-9033241	Peroxisomal protein import	−16.6
	R-HSA-9609507	Protein localization	−13.6
	GO:0046395	Carboxylic acid catabolic process	−10.2

Table 4 shows the most important GO biological processes, InterPro domains, and the KEGG pathways enriched in the IO&NS/Wnt network. The GO biological process enrichment analyses showed that this network was significantly associated with oxidoreductase, antioxidant and peroxidase activities, and β-catenin binding. InterPro enrichment analyses showed that heme peroxidase and glutathione peroxidase were the top terms. The KEGG path analysis showed that the network was highly associated with the Wnt signaling and NF-κB pathway, tryptophan metabolism, and illnesses including tuberculosis and Chagas disease.

**TABLE 4:** The gene ontology (GO) biological process terms, InterPro domains, and the kyoto encyclopedia of genes and genomes (KEGG) pathways associated with chronic fatigue spectrum disorders.

Path ID	Enrichment GO biological process	Observed	background	Strength	pFDR
GO:0016491	Oxidoreductase activity	49	726	0.95	1.29E-28
GO:0016209	Antioxidant activity	22	74	1.6	1.35E-23
GO:0004601	Peroxidase activity	17	41	1.74	6.98E-20
GO:0008013	Beta-catenin binding	16	86	1.39	4.55E-14
GO:0005515	Protein binding	102	7026	0.29	1.96E-13
GO:0042802	Identical protein binding	51	1896	0.55	1.96E-13
GO:0016641	Oxidoreductase activity, acting on the CH-NH2 group of donors, oxygen as acceptor	10	16	1.92	1.22E-12
GO:0050660	Flavin adenine dinucleotide binding	14	82	1.36	7.21E-12
GO:0020037	Heme binding	15	134	1.17	1.75E-10
GO:0003824	Catalytic activity	80	5486	0.29	8.35E-09
GO:0004602	Glutathione peroxidase activity	8	20	1.73	8.35E-09
**Path ID**	**Enrichment InterPro**	**Observed**	**background**	**Strength**	**pFDR**
IPR010255	Haem peroxidase superfamily	8	10	2.03	9.50E-09
IPR019791	Haem peroxidase, animal-type	8	10	2.03	9.50E-09
IPR037120	Haem peroxidase domain superfamily, animal type	8	10	2.03	9.50E-09
IPR000889	Glutathione peroxidase	7	8	2.07	4.98E-08
IPR029760	Glutathione peroxidase conserved site	7	8	2.07	4.98E-08
IPR002937	Amine oxidase	7	10	1.97	9.91E-08
IPR036188	FAD/NAD(P)-binding domain superfamily	10	56	1.38	2.41E-07
IPR029759	Glutathione peroxidase active site	6	7	2.06	9.34E-07
**Path ID**	**Enrichment KEGG**	**Observed**	**background**	**Strength**	**pFDR**
hsa04310	Wnt signaling pathway	18	154	1.19	3.25E-13
hsa05152	Tuberculosis	18	168	1.15	6.53E-13
hsa04064	NF-kappa B signaling pathway	15	101	1.3	1.30E-12
hsa00380	Tryptophan metabolism	11	41	1.55	1.43E-11
hsa04146	Peroxisome	13	79	1.34	1.43E-11
hsa05142	Chagas disease	14	99	1.27	1.43E-11
hsa05163	Human cytomegalovirus infection	18	218	1.04	1.43E-11
hsa01100	Metabolic pathways	40	1447	0.57	2.64E-11
hsa04520	Adherens junction	12	67	1.38	3.16E-11
hsa05010	Alzheimer disease	21	355	0.9	3.21E-11

**FIGURE 4:**
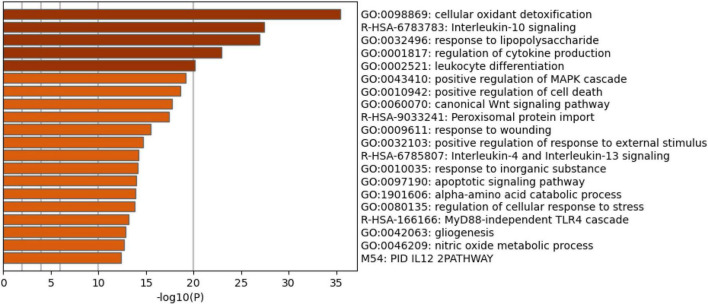
Heatmap of enriched ontology clusters showing the top 20 functions that were overexpressed in the network of patients with chronic fatigue spectrum disorders (accumulative hypergeometric *p*-values).

**FIGURE 5:**
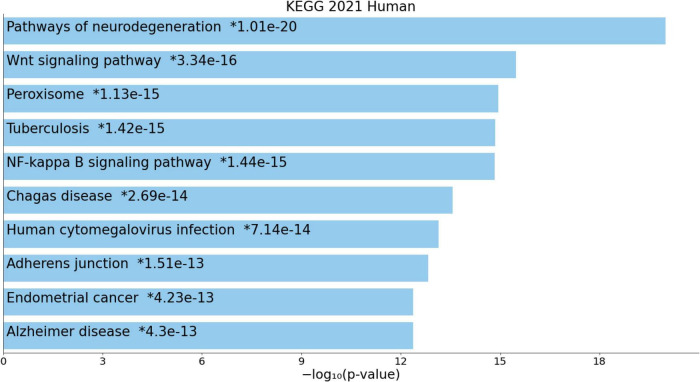
Heatmap (top 10) of the enriched Kyoto Encyclopedia of Genes and Genomes (KEGG) terms accumulated in the differently expressed proteins of the enlarged MultiOmics network of chronic fatigue spectrum disorders.

**FIGURE 6:**
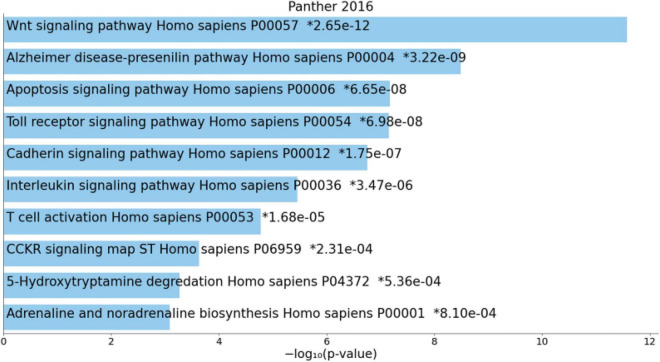
Heatmap (top 10) of enriched PANTHER terms accumulated in the differently expressed proteins of the enlarged MultiOmics network of chronic fatigue spectrum disorders. *significant after FDR p correction.

Figure 4 shows the enriched ontology clusters (using MetaScape) in the network with cellular oxidant detoxification, IL-10 signaling, and a response to LPS as the top terms. Figure 5 shows a heatmap (made using Enrichr and Appyter) with the top KEGG pathways that were overrepresented in the IO&NS/Wnt network, namely, pathways of neurodegeneration, Wnt and NF-κB signaling, and peroxisome. Figure 6 shows a heatmap with the PANTHER 2016 pathways, which were overrepresented in the network, namely, Wnt, apoptosis, TLR and cadherin pathways, and the Alzheimer’s disease pre-senilin pathway. TTRUST analysis showed that NF-κB (pFDR = 6.58E-23) and RELA (pFDR = 8.691E-21) were the most important transcriptional factors of the network followed at a large distance by SP1 (pFDR = 7.079E-13).

Table 5 shows the top 10 DOID annotations of an extended IO&NS/Wnt network (inBio Discover) including four intestinal disorders as the top 4 annotations, immune disorders, and cancer.

**TABLE 5:** Results of inBio discover annotation analysis with the top 10 and custom-made DOID annotations associated with chronic fatigue spectrum disorders.

DOID ID	Disease	Size	Overlap	Enrichment	p-value
DOID:77	Gastrointestinal system disease	2.3k	133/419	2.81	6.1E-30
DOID:5295	Intestinal disease	1.0k	82/419	3.86	1.3E-26
DOID:0050589	Inflammatory bowel disease	306	46/419	7.18	4.4E-26
DOID:00600180	Colitis	237	41/419	8.26	9.1E-26
DOID:2914	Immune system disease	1.9k	111/419	2.79	3.1E-24
DOID:612	Primary immunodeficiency disease	1.3k	87/419	3.12	6.4E-22
DOID:7	Disease of anatomical entity	7.3k	248/419	1.62	1.5E-21
DOID:417	Autoimmune disease	1.1k	75/419	3.36	1.2E-20
DOID:0050686	Organ system cancer	3.9k	161/419	1.99	2.8E-20
DOID:162	Cancer	4.2k	168/419	1.93	8.0E-20
**DOID ID**	**Selected annotations**	**Size**	**Overlap**	**Enrichment**	**p-value**
DOID:0097237	Cellular response to a toxic substance	77	23/419	14.26	1.2E-20
DOID:0042221	Response to chemical	2.4k	111/419	2.20	2.5E-16
DOID:0040085	Bacterial sepsis	13	9/419	33.05	4.7E-13
DOID:934	Viral infectious disease	664	43/419	3.23	4.3E-12
DOID:104	Bacterial infectious disease	268	27/419	4.81	1.7E-11
DOID:0042742	Response to bacterium	251	23/419	4.37	3.6E-9
DOID:0071219	Cellular response to a molecule of bacterial origin	80	13/419	7.76	1.1E-8
DOID:0050338	Primary bacterial infectious disease	219	20/419	4.36	4.08E-8
GO:0002237	Response to a molecule of bacterial origin	107	13/419	5.80	3.8E-7
DOID:0050339	Commensal bacterial infectious disease	46	9/419	9.34	4.0E-7

Electronic Supplementary File (ESF) 1 and ESF 2 show the results of enrichment and annotation analyses performed on the DEPs (or selected DEPs), as given in Figure 1.

## Discussion

### Networks and Subnetworks of Chronic Fatigue Spectrum Disorders

The first major finding of this study is that the PPI network of the DEPs and metabolics of CFAS-D show high connectivity and comprises two subnetworks, a first centered around *IO*&*NS* genes and a second centered around *Wnt/*β*-catenin* genes. The backbone of the master network comprises DEPs/metabolics, including NFKB1, CTNNB1, ALB, peroxides, NOS2, TNF, and IL-6, while in the giant network, many redox-related enzymes were predicted to be hubs, including endothelial NO synthase producing NO relaxing smooth muscle relaxation (NOS3), mitochondrial superoxide dismutase 2 (SOD2), and peroxiredoxin 6 (PRDX6) (catalyzes the reduction of hydrogen peroxides). It is important to note that without the delineation of the MPIs, one would have concluded that especially proinflammatory cytokine genes are the dominant forces in this network, whereas, in fact, the IO&NS subnetwork is more dominated by redox genes, NF-κB and IL-10.

CTNNB1, which belongs to the Wnt subnetwork, was another hotspot and additionally showed many interactions with genes in both the subnetworks indicating that this gene is a relevant switch linking both the subnetworks. Other relevant switches belonging to the immune subnetwork were ALB and IL-6, which showed many interactions with cluster 1 genes, but also with CTNNB1, DKK1, and AGRN. Hotspots and switches are new drugs targets because they govern and control the network and/or link the subnetworks (20). All in all, we may conclude that dysfunctions in the IO&NS and Wnt subnetworks underpin the pathophysiology of CFAS-D.

### Terms Overrepresented in the Immune-Inflammatory, Oxidative, and Nitrosative Multiomics Subnetwork

The second major finding of this study is that the top relevant functions and pathways in the first network revolve primarily around redox mechanisms, namely, cellular oxidant detoxification (and, thus, cellular detoxification and a cellular response to toxic substance), the hydrogen peroxide metabolic process, antioxidant activity, amine oxidase reactions, oxidoreductase activity, peroxidase activity, and heme and glutathione peroxidase. These findings indicate that increased oxidative stress (hydrogen peroxides) probably as a response to a toxic substance coupled with reduced antioxidant defenses (peroxidase, oxidase, oxidoreductase, and ALB) are the major pathways in CFAS-D. Indeed, many of the seed DEPs and metabolics show antioxidant properties, including ALB, GPX1, HP, CoQ10, DHEA, zinc, and EPA and pro-oxidant properties, including NFKB1, NOS2, PTGS2, and hydroperoxides. Moreover, the giant network based on seed DEPs and MPI-derived genes indicates that many redox genes are expected to participate in the CFAS-D network, including CAT, GPX2-9, SOD1, SOD2, LPO, LOX, MPO, NOS1, NOS3, and PRDX6.

Such findings agree with the evidence that CFAS-D is characterized by reduced antioxidant defenses and oxidative damage to lipids, proteins, DNA, and mitochondria as reviewed in (1, 3, 7, 15). Such damage in ME/CFS is accompanied by formation of immunogenic oxidative modified neoepitopes, which, in turn, may cause an IgM-mediated immune response against neoepitopes such as malondialdehyde and azelaic acid (18). The participation of NOS2 in the CFAS-D network is in agreement with our reports that ME/CFS is accompanied by increased nitrosative stress, which is a consequence of increased NO and superoxide production resulting in hypernitrosylation (18). In addition, also more recent articles indicate that increased nitro-oxidative stress and reduced antioxidant defenses contribute to idiopathic chronic fatigue, including increased reactive oxygen species (ROS), malondialdehyde, and F2-isoprostanes and lowered catalase and glutathione levels (21). In patients with CFAS-D, baseline thiobarbituric acid–reactive substances (TBARS) (an indicant of lipid peroxidation) are associated with exercise-induced pain (22). A recent review describes how oxidative stress may cause membranopathies, channelopathies, and mitochondrial dysfunctions, which, in turn, may interfere with intracellular energy processes and imbalances in signal conversion system (23). There are many reports showing that antioxidants may improve CFAS-D in animal models, including carvedilol, melatonin, *Withania somnifera*, quercetin and *Hypericum perforatum L* (24), curcumin (25), *Sarcodon imbricatus* (26), and many more.

The results of our network and enrichment analyses show that NFKB1 was not only one of the most important hotspots in the CFAS-D network, but also that NF-κB (p50–p52 unit) and RELA (NF-κB p65 unit or transcription factor p65) were the most important transcription factors controlling the network and that the NF-κB signaling pathway was one of the most important paths enriched in the network. NF-κB is a major transcriptional factor involved in the response to a vast array of stimuli, including bacterial or viral antigens, cytokines, free radicals, oxidized epitopes, and glutamate and is a transcriptional inducer of many genes, including cellular adhesion molecules, proinflammatory cytokines, chemokines, and growth, apoptosis and coagulation factors, and antioxidants and pro-oxidants (27–29). RELA is involved in the activation of NF-κB and stimulates NF-κB translocation to the nucleoplasm and by forming a RELA-NF-κB complex activates target gene expression (30). NF-κB p50, which is associated with RELA, and NF-κB p52 are major components of the canonical and non-canonical NF-κB signaling pathways, which both lead to target gene activation (29). Both the transcription factors are known to regulate different pathways that we observed to participate in the network, including the mitogen-activated protein kinase (MAPK) pathway (31) and ROS and Wnt/β-catenin pathways.

There are many intersections between reactive oxygen species (ROS) and NF-κB signaling. Indeed, ROS may modulate the NF-κB response leading to transcriptional activation of NF-κB-target antioxidant genes (e.g., *SOD*, *HO1*, *GPX1*, *TRX1*, and *TRX2*), which reduce ROS production, thereby promoting cell survival (29) and NF-κB-target pro-ROS genes, including *NOX2*, *COX2*, and *NOS2* (29, 32). It should be stressed that the effects of ROS on NF-κB are more than complex with stimulatory effects in the cytoplasm and inhibitory effects in the nucleus (33), while ROS may also oxidize NF-κB p50 leading to decreased DNA-binding capacity (34).

All in all, the results of our network and enrichment analysis and Maes et al. (35, 36) indicate that the complex cross-talks between NF-κB and ROS signaling are involved in the pathophysiology of CFAS-D. Moreover, recent studies show that increased NF-κB expression may be associated with central fatigue by modulating central nervous system genes and regulating immune-inflammatory processes, synaptic plasticity, and memory and exerting neurotoxic effects (35–37).

Our MCODE analysis revealed that the IL-10 anti-inflammatory signaling pathway, regulation of cytokine production, signaling by interleukins, and a response to LPS were a relevant molecular complex in the IO&NS subnetwork of CFAS-D. These findings agree with those of our previous studies showing increased IgA/IgM response to LPS of 6 different Gram-negative bacteria in CFAS-D, indicating an increased bacterial translocation (19). Previously, we have argued that part of the effects of translocated LPS on cytokine production could be explained by induction of the TLR signaling pathway, which also signals to NF-κB, which, in turn, may activate cytokine genes (38, 39).

Our enrichment analysis also showed an association between the IO&NS subnetwork and the lactoferrin (LTF) danger signal response pathway. LTF, an iron-binding glycoprotein, activates NF-κB via the TLR2/4 complexes and additionally RAGE and TREM-1 receptors (40). LTF functions as a first-line defense against injuries, either pathogenic or non-pathogenic, and controls cell homeostasis (40). Furthermore, LTF sequesters ROS (thereby attenuating tissue damage due to excessive IO&NS) and maintains intestinal integrity during endotoxemia (40). Our enrichment analysis shows that the proinflammatory TNF-related weak inducer of apoptosis (TWEAK) signaling pathway is another possible link between tissue injury and upregulation of NFKB1-associated DEPs/genes by activating the NF-κB pathways (41).

The NF-κB signaling pathway not only regulates the transcription of proinflammatory genes, including IFNG, IL-1β, and TNF, but also IL-10, which protects against an overzealous inflammatory response (42). IL-10 has negative immunoregulatory activities by inhibiting the production of M1 macrophage and T helper (Th)-1 cytokines, dendritic cells stimulating CD4 + T cells and Th-2 responses, and attenuates CD8 +, M1 macrophage, and Th-1 and Th-2 associated immunopathology (43, 44). It is important to note that our enrichment analysis stresses the importance of anti-inflammatory IL-10 signaling as a response to LPS and regulation of cytokine signaling, suggesting that a predominant IL-10 phenotype may occur in CFAS-D. Presuming that the latter are frequently triggered by a low/moderate pathogen (or LPS) virulence (1), we may expect that IL-10 suppresses the ongoing immune response, thereby contributing to long-term escape of pathogens from immune control and causing persistent or recurrent infections (1, 43). Therefore, immunosuppression, which is another hallmark of CFAS-D, may not only be explained by T-cell exhaustion through increased proinflammatory cytokine production (45), but also by an increased IL-10.

Finally, our network and enrichment analyses showed that the TRYCAT pathway may be involved in CFAS-D, although it is not a key component but rather a spin-off of the IO&NS response. Previously, it was reported that this pathway is highly strongly associated with somatization disorder, a psychiatric disease accompanied by psychosomatic symptoms but not necessarily by fatigue (46). The TRYCAT pathway acts as a redox regulator and is one of the major antioxidant systems, although some TRYCATs have pro-oxidant and neurotoxic activities (47). Thus, 3OHK, one of the metabolics in our MPI network, is one of the neurotoxic TRYCATS produced during activation of this pathway because of IO&NS activation. Kynurenine hydroxylase (KYNU) is, as IDO, an oxygen-consuming enzyme, which catabolizes kynurenine into 3OHK (STRING).

### Terms Overrepresented in the Wnt Multiomics Subnetwork

Our enrichment analyses revealed that the CFAS-D network was highly significantly associated with two major interrelated functions/pathways/domains, namely, the canonical Wnt/β-catenin pathway, T-cell factor (TCF)-dependent signaling in response to Wnt, degradation of β-catenin by the destruction complex, the β-catenin complex and DIX domain, and adherens junctions, cadherin prodomain, and cell–cell junctions. The DIX domains and axin, GSK-3, and Disheveled (Dvl) are key players in the β-catenin destruction complex, thereby determining the interaction of β-catenin with the transcription factor TCF and, thus, the expression of the Wnt target genes (48).

The β-catenin/E-cadherin complex is the key component of adherens junctions (AJs), which stabilizes cell–cell junctions, provides cell–cell adhesion, and binds β-catenin with the actin cytoskeleton (9, 49). Moreover, aberrations in the AJs lead to a breakdown of the tight junctions (TJs), another component of the paracellular route (50). β-catenin and occludin are key factors in both the AJs and TJs (paracellular pathway), including those of the blood–brain barrier (BBB) and gut barriers, whereas claudin-5 is more specific to the BBB and E-cadherin to the gut barrier (9).

Therefore, dysfunctions in AJs and TJs may cause aberrations in cell–cell adhesion and the actin cytoskeleton (51). Furthermore, degradation of the E-cadherin/β-catenin complexes may cause aberrations in intracellular signaling, the actin cytoskeleton, and the Wnt/β-catenin signaling pathway, a key component in cell homeostasis (52). Previously, we reported that increased levels of zonulin (prehaptoglobin-2) are strongly associated with CFAS-D in schizophrenia and explain, at least in part, the aberrations in TJs and AJs and consequent breakdown in the paracellular route (9). It should be added that aberrations in the paracellular route of the gut barrier are associated with an increased bacterial or LPS translocation (leaky gut) and, thus, activation of the IO&NS pathways (9). Moreover, increased ROS and NO production and proinflammatory pathways (TNF, IL-6, and IFNG) may damage the epithelial and endothelial TJs (53). Furthermore, hydrogen peroxides may redistribute β-catenin and E-cadherin from the paracellular route into the cell, thereby affecting the TJs and deplete occludin and damage the cytoskeleton as well (53). It should be added that virulence factors of many pathogenenic bacteria may exploit the Wnt/β-catenin pathway to alter the antibacterial immune response, including *Pseudomonas aeruginosa*, one of the bacteria involved in CFAS-D (19, 54).

Importantly, our MCODE analysis revealed another highly significant molecular complex in the CFAS-D network comprising the hydrogen peroxide metabolic process, amine oxidase reactions, and the β-catenin degradation pathway, indicating that interactions between those factors are involved. Importantly, dysregulated Wnt/β-catenin signaling may cause oxidative stress in pregnant mice with CFS (55). Nucleoredoxin (NRX), a thioredoxin-like protein, strongly inhibits Wnt/β-catenin binding and the expression of early genes, whereas H_2_O_2_ treatment stabilizes β-catenin and increases expression of Wnt genes (56). Increased ROS promotes intrinsic apoptosis and the consequent caspase-3 expression inhibits Wnt signaling in association with cleavage of E-cadherin (57). H_2_O_2_ not only inhibits Wnt/β-catenin signaling, but may also increase Wnt/β-catenin signaling (58). In fact, peroxides have a biphasic effect on Wnt signaling with an increase 20 min after activation and reduced signaling some hours later (57). Finally, β-catenin is a key regulator of the homeostatic cell response, which helps to repair the damage due to nitro-oxidative stress (59).

Furthermore, the NF-κB and Wnt pathways shows multiple cross-talks and negatively or positively regulate each other, thereby forming a mutual regulatory network (60). Wnt modulates the production of inflammatory cytokines and NF-κB signaling and bridges innate and adaptive immune pathways (61). On the other hand, increased levels of IL-6 and TNF-α may maintain increased Wnt signaling associated with low β-catenin, Disheveled, and axin levels (62). It should be stressed that our network analysis showed that the key seed DEPs of both the IO&NS (NFKB1) and Wnt (CTNBB1) pathways have significant physical interactions based on co-expression, experimental, and biochemical data (STRING), thereby also functioning as an anchor linking the IO&NS and Wnt subnetworks. All in all, it appears that the pathophysiology of CFAS-D may be characterized by aberrations in the intertwined cross-talks between ROS, NF-κB, and Wnt/β-catenin signaling.

Previously, we have explained how IO&NS and NF-κB and their associated pathways may explain the central and peripheral symptoms of CFAS-D (3, 15, 16, 23, 36, 63). This study shows that alterations in the Wnt pathway may contribute to these symptoms because this pathway regulates the homeostasis in self-renewing tissues and has additionally organ-specific effects. For example, the Wnt/β-catenin pathway is involved in BBB integrity, synapse assembly, synapse functions, synaptic plasticity, neurogenesis, white matter lesion remyelination, dopamine neuron neuronal survival protection, and regeneration, whereas aberrations in the Wnt/β-catenin pathway are associated with synaptic loss, BBB breakdown, and neurodegenerative disease, including Alzheimer’s and Parkinson’s disease (64–68).

The Wnt/β-catenin pathway is also involved in: (a) pain and neuropathic pain with Wnt inhibition improving pain (67, 69); (b) skeletal muscle dynamics, the neuromuscular synapse, and musculoskeletal functions, including the electrophysiologic properties of muscle cells (70, 71); and (c) intestinal functions, including epithelial homeostasis and integrity, the physiological proliferation of the transit-amplifying cells and differentiation of Paneth, goblet, and enteroendocrine cells, and the maintenance of mucosa and barrier functions (72–75). The Wnt/β-catenin pathway also plays a key role in autoimmunity as observed in rheumatoid arthritis (76, 77) and in response to bacterial infections and inflammation (78, 79).

## Conclusion

Aberrations in the cross-talks among redox, NF-κB, and Wnt signaling are predicted to be key pathways in CFAS-D, which are, therefore, associated with dysfunctions in multicellular organism homeostatic processes (including negative regulation of energy homeostasis and tissue maintenance). Disorders in the intertwined interactions between these systems may explain the broad spectrum of organs and dysfunctions that participate in CFAS-D (brain, musculoskeletal system, immune system, and gastrointestinal system). An important spin-off is increased IL-10 production, which may contribute to immunosuppression and recurrent or protracted infections and increased TRYCAT production may aggravate the neurotoxic effects of oxidative stress. Increased translocation of Gram-negative bacteria with increased LPS load is probably a major trigger factor, but also other bacterial infections, toxoplasmosis, viral infections (e.g., cytomegalovirus), cancer, and gastrointestinal, autoimmune, immune-inflammatory, neuroinflammatory, and neurodegenerative disorders appear to be associated with those pathways via activation of TLR/LTF/TWEAK signaling.

Future study should scrutinize the specific role of the NF-κB, ROS, and Wnt axis in CFAS-D. The cross-talks between these three pathways may also constitute new drug targets to treat CFAS-D. Given that the Wnt pathway shows many complex, negative and positive feedback loops interacting with redox systems and NK-κB signaling, manipulations of Wnt signaling and β-catenin (despite being a hub and master switch) appear to be very challenging. It may be more promising to simultaneously target the cross-talk among redox and NF-κB pathways. New knowledge on the precise aberrations in the Wnt pathway in association with ROS and NF-κB signaling may lead to more effective treatments by specifically targeting β-catenin, the β-catenin destruction complex, or the TCF transcription factor.

There are now many studies showing that targeting redox pathways with selected antioxidants may improve chronic fatigue-like symptoms in animal models of CFS and in chronic fatigue comorbid with neuroinflammatory disease (24, 26, 80, 81). In reserpine-induced zebrafish models of fibromyalgia (which is strongly associated with ME/CFS), treatment with hydroxytyrosol, a phenolic phytochemical that has antioxidant properties, regulates Wnt/β-catenin pathway activation and microglial activation in association with improving the behavioral deficits (82). Administration of Ashwagandha, which has antioxidant properties, to rats with thioacetamide-induced hepatic encephalopathy improves neurocognitive deficits in association with attenuation of NF-κB/MAPK pathways and induction of antioxidant responses via Nrf-2 (83). In addition, as reviewed in the Introduction, many studies indicate that administration of diverse antioxidants improve CFAS-D in animal models (24–26). Since GSK-3B is one of the proteins involved in the PPI network, it could be argued that inhibiting GSK-3B is another possible target to treat the condition and that this could be achieved using different GSK-3 inhibitors including lithium (84). Moreover, some studies showed that GSK-3 inhibition using low-dose lithium may augment muscle force production (85). Nevertheless, GSK-3B is not a key gene in the PPI network and, thus, GSK-3 inhibition will probably yield minimal effects. As such, animal models support that interactions among redox, Wnt/β-catenin and NF-κB pathways participate in CFS-like behaviors and that targeting IO&NS pathways may improve these behaviors.

**FIGURE 7:**
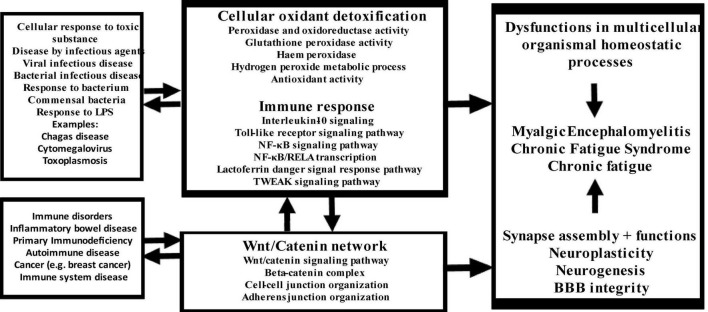
Summary of the findings in this study. LPS: lipopolysaccharides, NF-κB: nuclear factor-κB, RELA: transcription factor p65, TWEAK: TNF-related weak inducer of apoptosis.

The new model of CFAS-D proposed here is shown in Figure 7. Disorders in the cross-talks among these three key pathways mediate the effects of a variety of trigger factors in the onset of CFAS-D. These findings also support the theory that once CFAS-D is present, the abovementioned pathways may increase morbidity and even mortality of IO&NS-associated medical disorders through the detrimental effects of disorders in redox, NF-κB, and Wnt axis (24, 26, 86–88). The model also explains that the acute phase of inflammatory conditions may lead to CFAS-D via disorders in this axis, oxidative damage and the neurotoxic effects of LPS, proinflammatory cytokines, and TRYCATs. Since the biomarkers included in this study were extracted from studies on ME/CFS and CF-like symptoms in comorbid disorders with duration > 6 months, we may conclude that after resolution of acute inflammation, CAFS-D symptoms are maintained by continued aberrations in the redox, NF-κB, and Wnt axis, increased IL-10 production, and increasing oxidative damage, including secondary autoimmune responses and nitrosylation.

## Author Contributions

MM designed the study and performed the network, enrichment, and annotation analyses. All authors contributed to interpretation of the data, writing of the manuscript, and participated in the manuscript.

## Conflict of Interest

The authors declare that the research was conducted in the absence of any commercial or financial relationships that could be construed as a potential conflict of interest.

## Publisher’s Note

All claims expressed in this article are solely those of the authors and do not necessarily represent those of their affiliated organizations, or those of the publisher, the editors and the reviewers. Any product that may be evaluated in this article, or claim that may be made by its manufacturer, is not guaranteed or endorsed by the publisher.

## Footnotes

1
https://www.genome.jp/kegg/

2
https://string-db.org

3
https://cytoscape.org

4
www.omicsnet.ca

5
https://www.ebi.ac.uk/intact/

6
https://www.grnpedia.org/trrust/v1/

7
https://maayanlab.cloud/Enrichr/

8
https://metascape.org

9
https://inbio-discover.com

10
https://reactome.org/

11
pantherdb.org

12
ebi.ac.uk

13
disease-ontology.org
