# Supplementary material for: Aberrations in the Cross-Talks Among Redox, Nuclear Factor-κB, and Wnt/β-Catenin Pathway Signaling Underpin Myalgic Encephalomyelitis and Chronic Fatigue Syndrome
Source: Front Psychiatry. 2022 May 6;13:822382. doi: 10.3389/fpsyt.2022.822382 (PMC9120845; doi:10.3389/fpsyt.2022.822382)
Supplement: Supplementary file 1 [file Data_Sheet_1.pdf]

ELECTRONIC SUPPLEMENTARY FILE (ESF) 1

Aberrations in the cross-talks among redox, nuclear factor-κB and Wnt/catenin pathway signaling underpin Myalgic Encephalomyelitis and chronic fatigue syndrome: a review and new hypothesis based on results of network, enrichment and annotation analyses.

Michael Maes, M.D., Ph.D. [a,b,c], Marta Kubera, [d] Magdalena Kotańska, Ph.D [e]

[a] Department of Psychiatry, Faculty of Medicine, Chulalongkorn University, Bangkok, Thailand

[b] Department of Psychiatry, Medical University of Plovdiv, Plovdiv, Bulgaria

[c] IMPACT Strategic Research Center, Deakin University, Geelong, Australia

[d] Laboratory of Immunoendocrinology, Department of Experimental Neuroendocrinology, Maj Institute of Pharmacology, Polish Academy of Sciences, 12 Smętna St., 31-343 Crakow, Poland

[e] Department of Pharmacological Screening, Jagiellonian University, Medical College, Medyczna 9, PL 30-688 Cracow, Poland

Enrichment analysis in all DEPs of CFAS-D shown in Figure 1.

ESF 1, Table 1 displays the results of MCODE analysis employing KEGG, WikiPaths, GO biological and molecular, REACTOME and PANTHER performed on the first-order network built using all seed genes. We observed three significant molecular complexes: the first represents IL10 signaling and signaling by interleukins; the second reflects canonical Wnt signaling and cell-cell signaling by Wnt; and the third represents TCF-dependent signaling in response to Wnt and Wnt signaling.

ESF 2, Figure 1 shows the enriched ontology term clusters in the first order network build using all genes (the network is constructed and visualized using MetaScape and Cytoscape, v3.1.2). This figure shows that the immune and Wnt subnetworks are strongly intertwined as well as the MAPK cascade and that these pathways are interconnected with multicellular organismal homeostasis.

ESF 2, Figure 2 shows the top-20 terms which were over-represented in the first order network built using all genes. This bar graph shows that besides cytokine (especially IL10) and Wnt signaling also positive regulation of the MAPK cascade is a significant path.

ESF 1, Table 2 shows the GO biological process and WikiPathways associated with CFAS-D. The top GO annotation terms were: the cell surface receptor signaling pathway, positive regulation of response to stimulus, and positive regulation of cell communication. The top WikiPathway names associated with CFAS-D were Alzheimer's disease, TNF-related weak inducer of apoptosis (TWEAK) signaling pathway, LTF danger signal response pathway, TLR signaling pathway, and miRNAs involvement in the immune response in sepsis

Enrichment analysis on the immune subnetwork genes of CFASD shown in Figure 1.

ESF 2, Figure 3 shows the enriched ontology term clusters in the immune subnetwork of CFAS-D and that cytokine (in particular IL10, but also IL4 and IL13) signaling, a response to LPS or an external stimulus were strongly interacting pathways.

ESF 2, Figure 4 shows the top WikiPaths which were over-represented in the immune subnetwork, namely the LTF danger signal response pathway and the TLR pathway. **ESF 2, Figure 5** shows a bar graph with the top-10 BioCarta terms which are over-represented in the immune subnetwork, namely NFKB pathway, anti-inflammatory IL10, IL1R, TNFR1 and ceramide signaling. **ESF 2, Figure 6** shows the top-10 KEGG annotations including Chagas disease, tuberculosis, CMV infection, toxoplasmosis, and viral protein interactions with cytokine and cytokine receptor.

Enrichment analysis on the Wnt subnetwork genes of CFAS-D shown in Figure 1.

ESF 2, Figure 7 and 8 shows the enriched ontology term clusters in the Wnt subnetwork of CFAS-D. Besides Wnt/catenin-associated paths, these also include diseases of signal transduction, signaling by Wnt in cancer, PID PS1 pathway and cell-junction organization. **ESF 2, Figure 9** shows the WikiPathways which were statistically over-represented in the Wnt/catenin subnetwork, including involvement of this pathway in colorectal cancer, leukemia, endometrial and breast cancer, ect. The bar graph shown in **ESF 2, Figure 10** shows the top-10 InterPro domains which were enriched in the Wnt subnetwork, including the cadherin prodomain and cytoplasmic domain, and the DIX domain. **ESF 2, Figure 11** shows a bar graph with top-10 enriched GO cellular components, including the catenin complex and adherens and cell-cell junctions. TTRUST enrichment analysis showed that NFKB1 (pFDR=2.911E-25) and RELA (pFDR=1.014E-22) were the two most important transcriptional factors in this network.

Annotation analyses and functional categorization of the PPI network and selected genes

ESF 2, Figures 12 and 13 depict the hierarchical structure of GO terms and the hubs and master regulatory transcription factor (ESF 2, Figure 12), downregulated seed genes (**ESF 2, Figure 13A**), seed genes of the Wnt/catenin pathway subnetwork (**ESF 2, Figure 13B**), and the major hotspots in the STRING enlarged networks (**ESF 2, Figure 13C**). Thus, ESF 2, Figure 12 shows that the GO functions which are regulated by NFKB1, CTNNB1, TNF and IL6. ESF 2, Figure 13A shows that the downregulated genes are associated with cellular oxidant detoxification and detoxification in general. ESF 2, Figure 13B shows that the seed genes of the Wnt subnetwork are associated with synapse organization and cell-cell signaling, whereas the major hotspots of the enlarged network (ESF 2, Figure 13C) are associated with a variety of processes including smooth muscle cell proliferation, regulation of DNA metabolic and apoptotic processes, and response to lipids, steroid hormones.

ESF 2, Figure 14 displays an extended network constructed with inBio Discover showing 3 of these DOID annotations (DOID:77, DOID:2914, and DOID:612) and 2 other DOID annotations (all <1.0E-6), namely autoimmune disease of musculoskeletal system and disease by an infectious agent. Table 6 show the results of annotation analyses with selected GO terms (with keywords: response to .., viral and bacterial).

ESF 1, Table 1. Results of Molecular Complex Detection (MCODE) analysis performed on the differently expressed proteins (DEPs) of chronic fatigue spectrum disorders.

MCODE Components	GO ID	Biological term	Log10 (p)
All DEPs, MCODE1	R-HSA-6783783	Interleukin-10 signaling	-32.5
	R-HSA-449147	Signaling by Interleukins	-31.9
	R-HSA-1280215	Cytokine signaling in immune system	-30.2
All DEPs, MCODE2	GO:0060070	Canonical Wnt signaling pathway	-12.4
	GO:0016055	Wnt signaling pathway	-10.9
	GO:0198738	Cell-cell signaling by Wnt	-10.9
All DEPs, MCODE3	R-HSA-201681	TCF dependent signaling in response to Wnt	-5.3
	GO:0060070	Canonical Wnt signaling pathway	-4.9
	R-HSA-195721	Signaling by WNT	-4.8

ESF 1, Table 2. Go Biological Process and WikiPathway terms associated with the network of chronic fatigue spectrum disorders (CFAS-D).

**Path ID**	**GO biological process names associated with CFAS-D**	**Observed**	**background**	**Strength**	**pFDR**
GO:0007166	Cell surface receptor signaling pathway	63	2325	0.77	1.23E-32
GO:0048584	Positive regulation of response to stimulus	60	2257	0.76	4.05E-30
GO:0048583	Regulation of response to stimulus	72	4114	0.58	3.43E-28
GO:0010647	Positive regulation of cell communication	52	1823	0.79	3.87E-26
GO:0023056	Positive regulation of signaling	52	1831	0.79	3.87E-26
GO:0009967	Positive regulation of signal transduction	50	1654	0.81	4.58E-26
GO:0042221	Response to chemical	71	4333	0.55	5.73E-26
GO:0009893	Positive regulation of metabolic process	68	3893	0.57	9.50E-26
GO:0070887	Cellular response to chemical stimulus	61	2919	0.65	1.05E-25
GO:0048522	positive regulation of cellular process	77	5579	0.47	2.79E-25
**Path ID**	**WikiPathway names associated with CFAS-D**	**Observed**	**background**	**Strength**	**pFDR**
WP5124	Alzheimers disease	20	255	1.23	1.94E-15
WP2036	TNF-related weak inducer of apoptosis (TWEAK) signaling pathway	11	42	1.75	4.25E-13
WP5039	SARS-CoV-2 innate immunity evasion and cell-specific immune response	12	66	1.59	6.76E-13
WP4478	LTF danger signal response pathway	9	19	2.01	1.46E-12
WP75	Toll-like receptor signaling pathway	13	103	1.43	2.01E-12
WP4329	miRNAs involvement in the immune response in sepsis	10	37	1.76	3.05E-12
WP4155	Endometrial cancer	11	63	1.57	8.07E-12
WP3658	Wnt/beta-catenin signaling pathway in leukemia	9	26	1.87	8.09E-12
WP231	TNF-alpha signaling pathway	12	92	1.45	9.77E-12
WP4258	lncRNA in canonical Wnt signaling and colorectal cancer	12	93	1.44	9.99E-12

ESF 1. References

Asad HN, Al-Hakeim H, Moustafa SR, Maes M. A causal-pathway phenotype of chronic fatigue syndrome due to hemodialysis. Submitted.

Al-Hakeim H, Moustafa SR, Maes M. Biomarkers of chronic fatigue and depression in patients with rheumatoid arthritis: a nomothetic network analysis. Submitted.

Al-Jassas H, Al-Hakeim H, Maes M. Intersections between Pneumonia, Lowered Oxygen Saturation Percentage and Immune Activation Mediate Depression, Anxiety and Chronic Fatigue Syndrome-like Symptoms due to COVID-19: A Nomothetic Network Approach. Preprints 2021, 2021060362 (doi: 10.20944/preprints202106.0362.v1).

Maes M, Kubera M, Stoyanova K, Leunis JC. The reification of the clinical diagnosis of myalgic encephalomyelitis / chronic fatigue syndrome (ME/CFS) as an immune and oxidative stress disorder: construction of a data-driven nomothethic network and exposure of ME/CFS subgroups. Curr Top Med Chem. 2021 Jul 27. doi:10.2174/1568026621666210727170147. Epub ahead of print. PMID: 34315375.

Mousa RF, Al-Hakeim HK, Alhaideri A, Maes M. Chronic fatigue syndrome and fibromyalgia-like symptoms are an integral component of the phenome of schizophrenia: neuro-immune and opioid system correlates. Metab Brain Dis. 2021 Jan;36(1):169-183. doi: 10.1007/s11011-020-00619-x. Epub 2020 Sep 23. PMID:32965599.

Almulla AF, Al-Hakeim HK, Abed MS, Carvalho AF, Maes M. Chronic fatigue and fibromyalgia symptoms are key components of deficit schizophrenia and are strongly associated with activated immune-inflammatory pathways. Schizophr Res. 2020 Aug;222:342-353. doi: 10.1016/j.schres.2020.05.003. Epub 2020 May 26. PMID: 32467068.

Al-Hakeim HK, Al-Issa AAR, Maes M. Serum agrin and talin are increased in major depression while agrin and creatine phosphokinase are associated with chronic fatigue and fibromyalgia symptoms in depression. Metab Brain Dis. 2020 Jan;35(1):225-235. doi: 10.1007/s11011-019-00506-0. Epub 2019 Nov 16. PMID:31734845.

Kanchanatawan B, Thika S, Sirivichayakul S, Carvalho AF, Geffard M, Maes M. In Schizophrenia, Depression, Anxiety, and Physiosomatic Symptoms Are Strongly Related to Psychotic Symptoms and Excitation, Impairments in Episodic Memory, and Increased Production of Neurotoxic Tryptophan Catabolites: a Multivariate and Machine Learning Study. Neurotox Res. 2018 Apr;33(3):641-655. doi: 10.1007/s12640-018-9868-4. Epub 2018 Jan 29. PMID: 29380275.

Kanchanatawan B, Sirivichayakul S, Thika S, Ruxrungtham K, Carvalho AF, Geffard M, Anderson G, Noto C, Ivanova R, Maes M. Physio-somatic symptoms in schizophrenia: association with depression, anxiety, neurocognitive deficits and the tryptophan catabolite

pathway. Metab Brain Dis. 2017 Aug;32(4):1003-1016. doi: 10.1007/s11011-017-9982-7. Epub 2017 Mar 3. PMID: 28258445.

Maes M, Bosmans E, Kubera M. Increased expression of activation antigens on CD8+ T lymphocytes in Myalgic Encephalomyelitis/chronic fatigue syndrome: inverse associations with lowered CD19+ expression and CD4+/CD8+ ratio, but no associations with (auto)immune, leaky gut, oxidative and nitrosative stress biomarkers. Neuro Endocrinol Lett. 2015;36(5):439-46. PMID: 26707044.

Maes M. A new case definition of Neuro-Inflammatory and Oxidative Fatigue (NIOF), a neuroprogressive disorder, formerly known as chronic fatigue syndrome or Myalgic Encephalomyelitis: results of multivariate pattern recognition methods and external validation by neuro-immune biomarkers. Neuro Endocrinol Lett. 2015;36(4):320-9. PMID: 26454487.

Maes M, Leunis JC, Geffard M, Berk M. Evidence for the existence of Myalgic Encephalomyelitis/Chronic Fatigue Syndrome (ME/CFS) with and without abdominal discomfort (irritable bowel) syndrome. Neuro Endocrinol Lett. 2014;35(6):445-53. PMID: 25433843.

Morris G, Maes M. Oxidative and Nitrosative Stress and Immune-Inflammatory Pathways in Patients with Myalgic Encephalomyelitis (ME)/Chronic Fatigue Syndrome (CFS). Curr Neuropharmacol. 2014 Mar;12(2):168-85. doi: 10.2174/1570159X11666131120224653. PMID: 24669210; PMCID: PMC3964747.

Maes M, Ringel K, Kubera M, Anderson G, Morris G, Galecki P, Geffard M. In myalgic encephalomyelitis/chronic fatigue syndrome, increased autoimmune activity against 5-HT is associated with immuno-inflammatory pathways and bacterial translocation. J Affect Disord. 2013 Sep 5;150(2):223-30. doi: 10.1016/j.jad.2013.03.029. Epub 2013 May 10. PMID: 23664637.

Maes M, Twisk FN, Ringel K. Inflammatory and cell-mediated immune biomarkers in myalgic encephalomyelitis/chronic fatigue syndrome and depression: inflammatory markers are higher in myalgic encephalomyelitis/chronic fatigue syndrome than in depression. Psychother Psychosom. 2012;81(5):286-95. doi: 10.1159/000336803. Epub 2012 Jul 20. PMID: 22832503.

Maes M, Twisk FN, Kubera M, Ringel K. Evidence for inflammation and activation of cell-mediated immunity in Myalgic Encephalomyelitis/Chronic Fatigue Syndrome (ME/CFS): increased interleukin-1, tumor necrosis factor-α, PMN-elastase, lysozyme and neopterin. J Affect Disord. 2012 Feb;136(3):933-9. doi: 10.1016/j.jad.2011.09.004. Epub 2011 Oct 4. PMID: 21975140.

Maes M, Mihaylova I, Kubera M, Uytterhoeven M, Vrydags N, Bosmans E. Lower whole blood glutathione peroxidase (GPX) activity in depression, but not in myalgic encephalomyelitis / chronic fatigue syndrome: another pathway that may be associated with coronary artery disease and neuroprogression in depression. Neuro Endocrinol Lett. 2011;32(2):133-40. PMID: 21552194.

Maes M, Kubera M, Uytterhoeven M, Vrydags N, Bosmans E. Increased plasma peroxides as a marker of oxidative stress in myalgic encephalomyelitis/chronic fatigue syndrome (ME/CFS). Med Sci Monit. 2011 Apr;17(4):SC11-5. doi: 10.12659/msm.881699. PMID: 21455120; PMCID: PMC3539515.

Maes M, Mihaylova I, Kubera M, Uytterhoeven M, Vrydags N, Bosmans E. Coenzyme Q10 deficiency in myalgic encephalomyelitis/chronic fatigue syndrome (ME/CFS) is related to fatigue, autonomic and neurocognitive symptoms and is another risk factor explaining the early mortality in ME/CFS due to cardiovascular disorder. Neuro Endocrinol Lett. 2009;30(4):470-6. PMID: 20010505.

Maes M, Mihaylova I, Bosmans E. Not in the mind of neurasthenic lazybones but in the cell nucleus: patients with chronic fatigue syndrome have increased production of nuclear factor kappa beta. Neuro Endocrinol Lett. 2007 Aug;28(4):456-62. PMID: 17693979.

Maes M, Mihaylova I, Kubera M, Bosmans E. Not in the mind but in the cell: increased production of cyclo-oxygenase-2 and inducible NO synthase in chronic fatigue syndrome. Neuro Endocrinol Lett. 2007 Aug;28(4):463-9. PMID: 17693978.

Mihaylova I, DeRuyter M, Rummens JL, Bosmans E, Maes M. Decreased expression of CD69 in chronic fatigue syndrome in relation to inflammatory markers: evidence for a severe disorder in the early activation of T lymphocytes and natural killer cells. Neuro Endocrinol Lett. 2007 Aug;28(4):477-83. PMID: 17693977.

Maes M, Mihaylova I, Leunis JC. In chronic fatigue syndrome, the decreased levels of omega-3 poly-unsaturated fatty acids are related to lowered serum zinc and defects in T cell activation. Neuro Endocrinol Lett. 2005 Dec;26(6):745-51. PMID: 16380690.

Maes M, Mihaylova I, De Ruyter M. Lower serum zinc in Chronic Fatigue Syndrome (CFS): relationships to immune dysfunctions and relevance for the oxidative stress status in CFS. J Affect Disord. 2006 Feb;90(2-3):141-7. doi: 10.1016/j.jad.2005.11.002. Epub 2005 Dec 9. PMID: 16338007.

Maes M, Mihaylova I, De Ruyter M. Decreased dehydroepiandrosterone sulfate but normal insulin-like growth factor in chronic fatigue syndrome (CFS): relevance for the inflammatory response in CFS. Neuro Endocrinol Lett. 2005 Oct;26(5):487-92. PMID: 16264414.
