# Supplementary material for: Aberrations in the Cross-Talks Among Redox, Nuclear Factor-κB, and Wnt/β-Catenin Pathway Signaling Underpin Myalgic Encephalomyelitis and Chronic Fatigue Syndrome
Source: Front Psychiatry. 2022 May 6;13:822382. doi: 10.3389/fpsyt.2022.822382 (PMC9120845; doi:10.3389/fpsyt.2022.822382)
Supplement: Supplementary file 2 [file Data_Sheet_2.PDF]

Aberrations in the cross-talks among redox, nuclear factor-κB and Wnt/catenin pathway signaling underpin Myalgic Encephalomyelitis and chronic fatigue syndrome: a review and new hypothesis based on results of network, enrichment and annotation analyses.

Michael Maes, M.D., Ph.D. [a,b,c], Marta Kubera [d], Magdalena Kotańska, Ph.D [c]

[a] Department of Psychiatry, Faculty of Medicine, Chulalongkorn University, Bangkok, Thailand

[b] Department of Psychiatry, Medical University of Plovdiv, Plovdiv, Bulgaria

[c] IMPACT Strategic Research Center, Deakin University, Geelong, Australia

[d] Laboratory of Immunoendocrinology, Department of Experimental Neuroendocrinology, Maj Institute of Pharmacology, Polish Academy of Sciences, 12 Smętna St., 31-343 Crakow, Poland

[e] Department of Pharmacological Screening, Jagiellonian University, Medical College, Medyczna 9, PL 30-688 Cracow, Poland

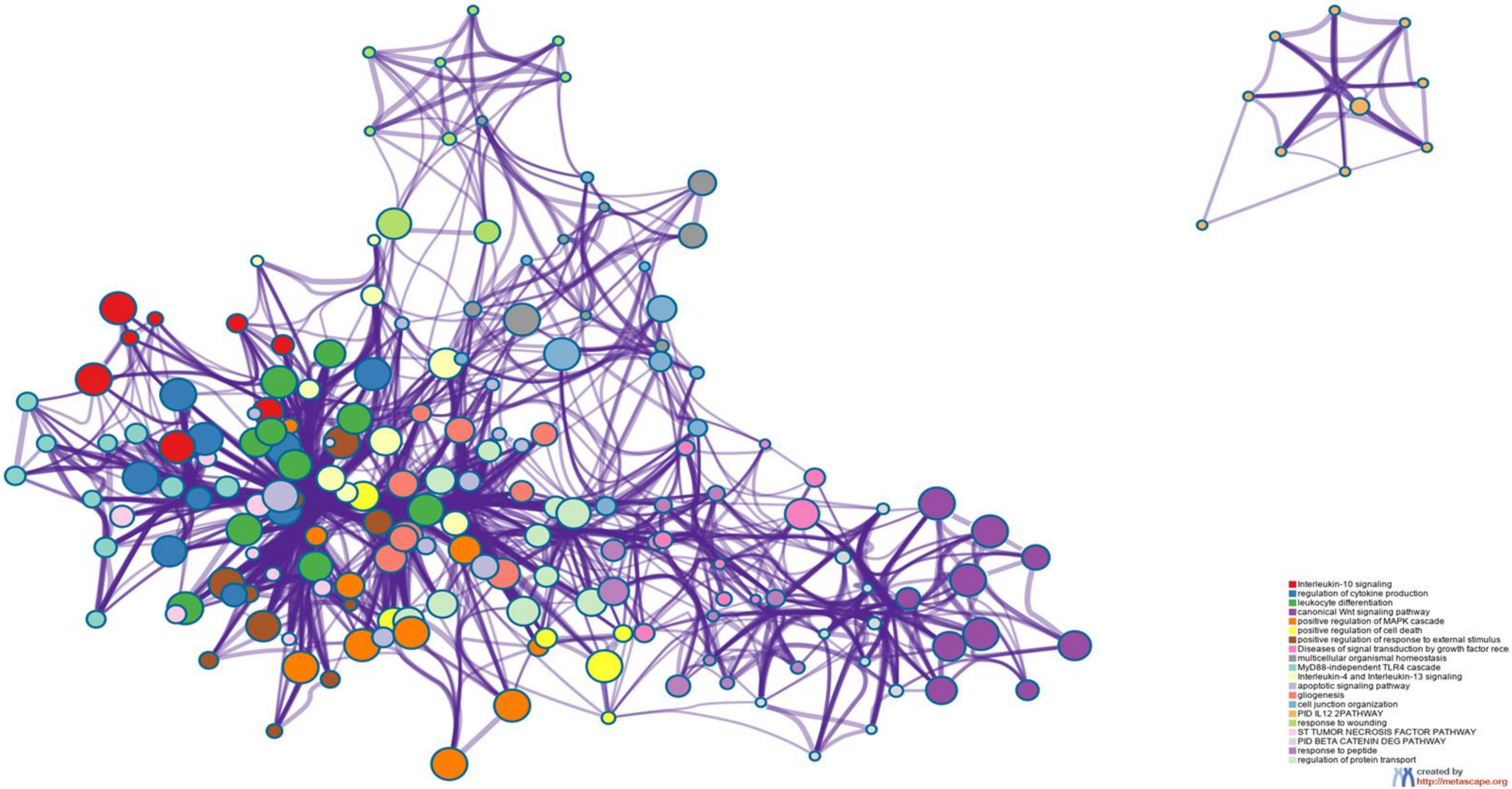
Interleukin-10 signaling
regulation of cytokine production
leukocyte differentiation
canonical Wnt signaling pathway
positive regulation of MAPK cascade
positive regulation of cell death
positive regulation of response to external stimulus
Diseases of signal transduction by growth factor rece
multicellular organismal homeostasis
MyD88-independent TLR4 cascade
Interleukin-4 and Interleukin-13 signaling
apoptotic signaling pathway
gliogenesis
cell junction organization
PID IL12 2PATHWAY
response to wounding
ST TUMOR NECROSIS FACTOR PATHWAY
PID BETA CATENIN DEG PATHWAY
response to peptide
regulation of protein transport
created by
http://metascape.org

ESF 2, Figure 1. Enriched ontology term clusters in the chronic fatigue spectrum disorders network. Terms are represented by a circle node, with the colors representing cluster identity and their size reflecting the number of input genes. One term is used to describe the clusters (colored labels). The thickness of the (bundled) edges linking the terms reflects the similarity score (threshold is > 0,3). The network is constructed and visualized using MetaScape and Cytoscape (v3.1.2)

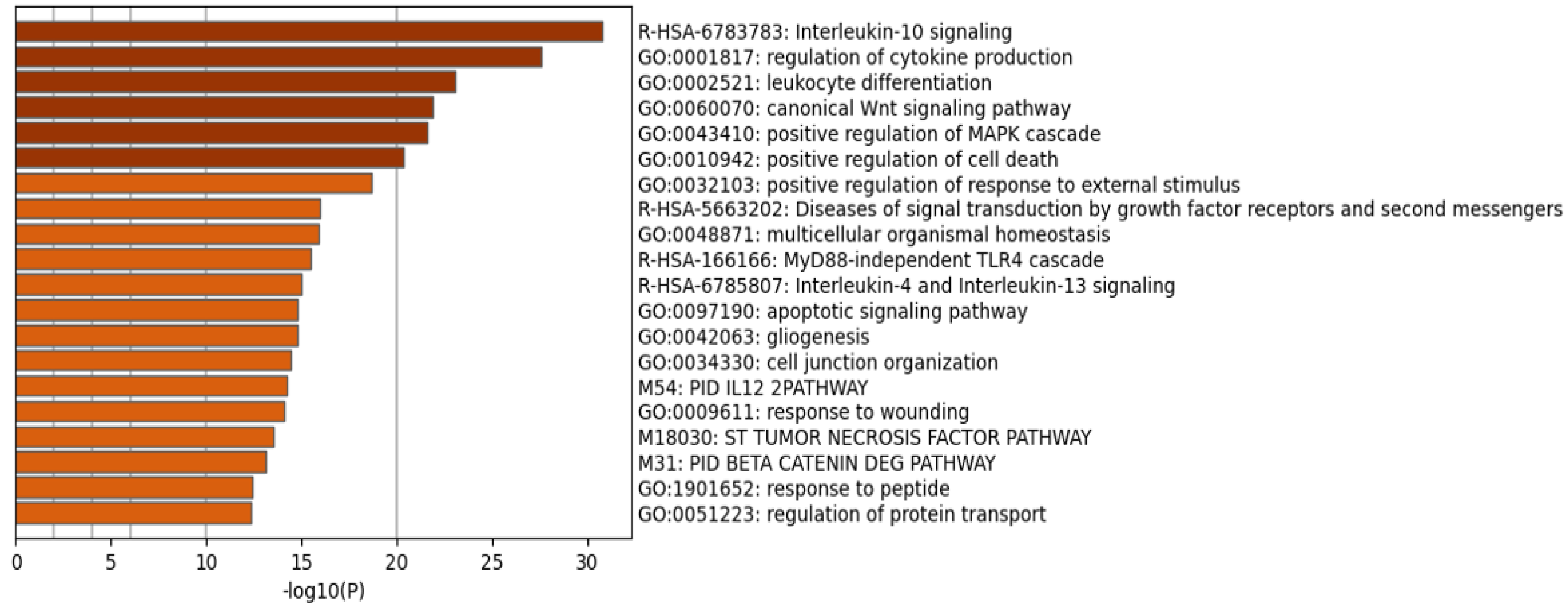

ESF 2, Figure 2. Heatmap of enriched ontology clusters showing the top-20 functions that were overexpressed in the expanded network of patients with chronic fatigue spectrum disorders (accumulative hypergeometric p-values)

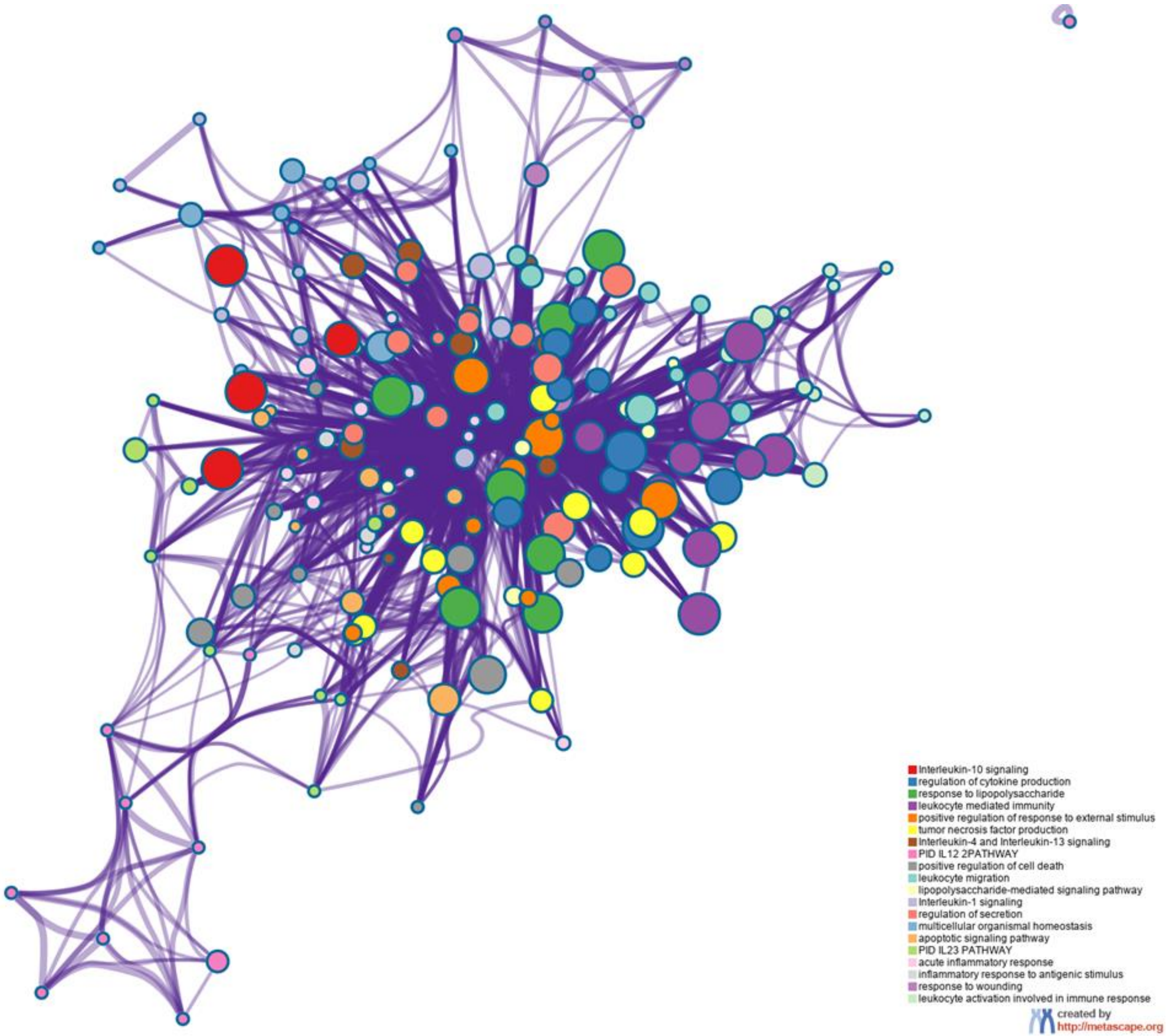
Interleukin-10 signaling
regulation of cytokine production
response to lipopolysaccharide
leukocyte mediated immunity
positive regulation of response to external stimulus
tumor necrosis factor production
Interleukin-4 and Interleukin-13 signaling
PID IL12 2PATHWAY
positive regulation of cell death
leukocyte migration
lipopolysaccharide-mediated signaling pathway
Interleukin-1 signaling
regulation of secretion
multicellular organismal homeostasis
apoptotic signaling pathway
PID IL23 PATHWAY
acute inflammatory response
inflammatory response to antigenic stimulus
response to wounding
leukocyte activation involved in immune response
created by
http://metascape.org

ESF 2, Figure 3. Enriched ontology term clusters in the immune subnetwork of chronic fatigue spectrum disorders. Terms are represented by a circle node, with the colors representing cluster identity and their size reflecting the number of input genes. One term is used to describe the clusters (colored labels). The thickness of the (bundled) edges linking the terms reflects the similarity score (threshold is > 0.3). The network is constructed and visualized using MetaScape and Cytoscape (v3.1.2)

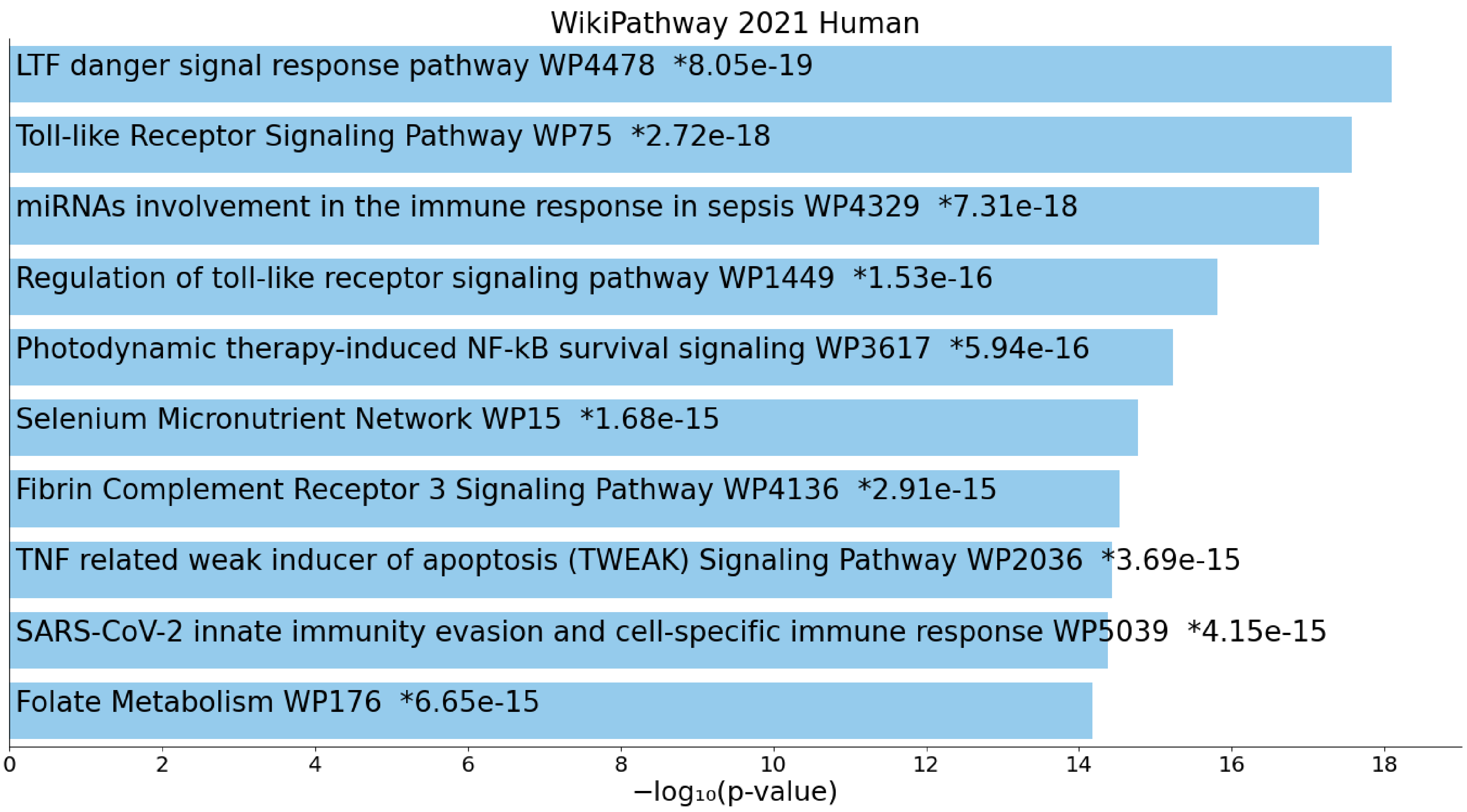

ESF 2, Figure 4. Heatmap (top-10) of the enriched WikiPathway terms accumulated in the differently expressed proteins of the immune subnetwork of chronic fatigue spectrum disorders

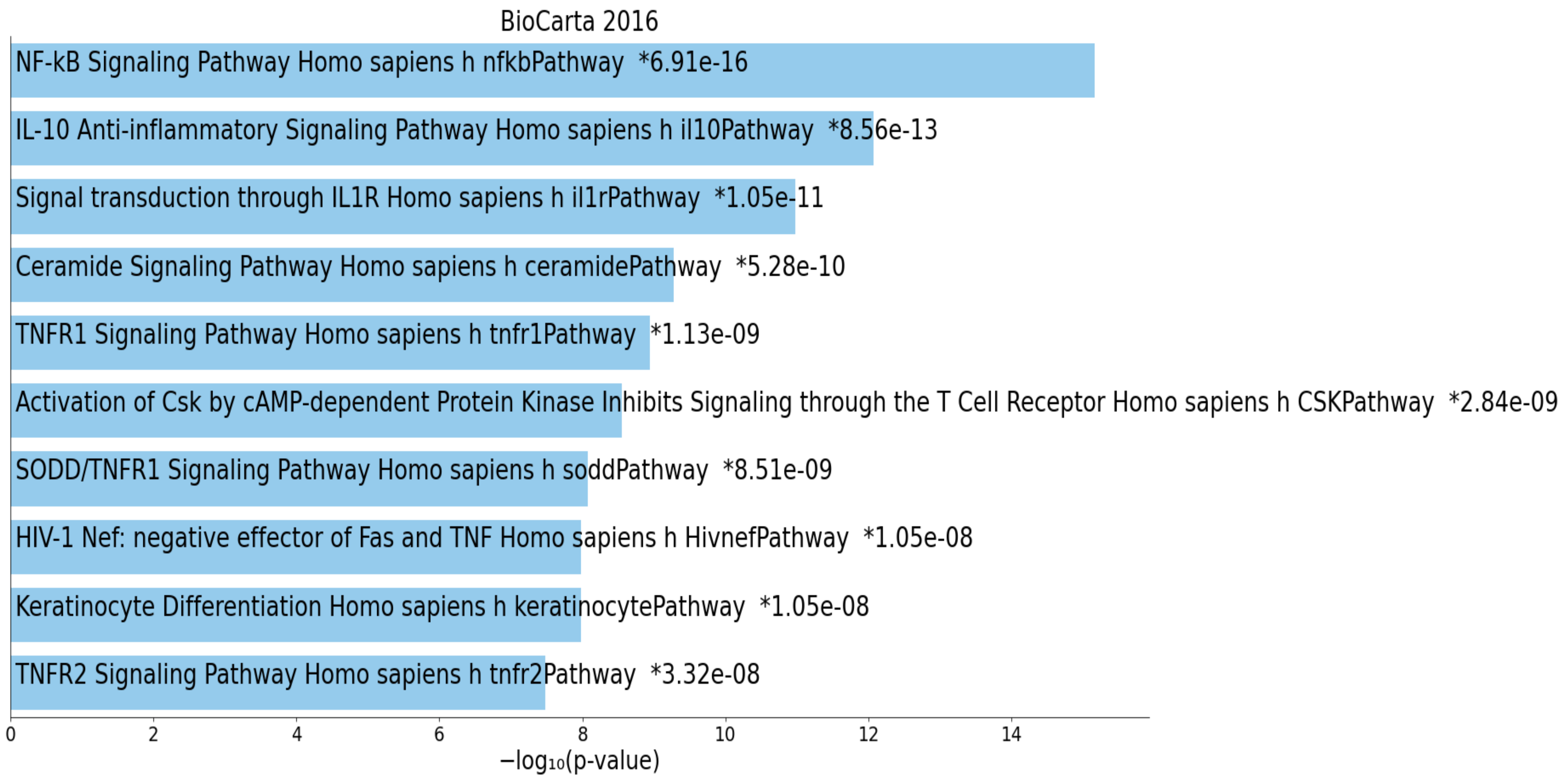

ESF 2, Figure 5. Heatmap (top-10) of the BioCarta terms accumulated in the differently expressed proteins of the expanded immune subnetwork of chronic fatigue spectrum disorders

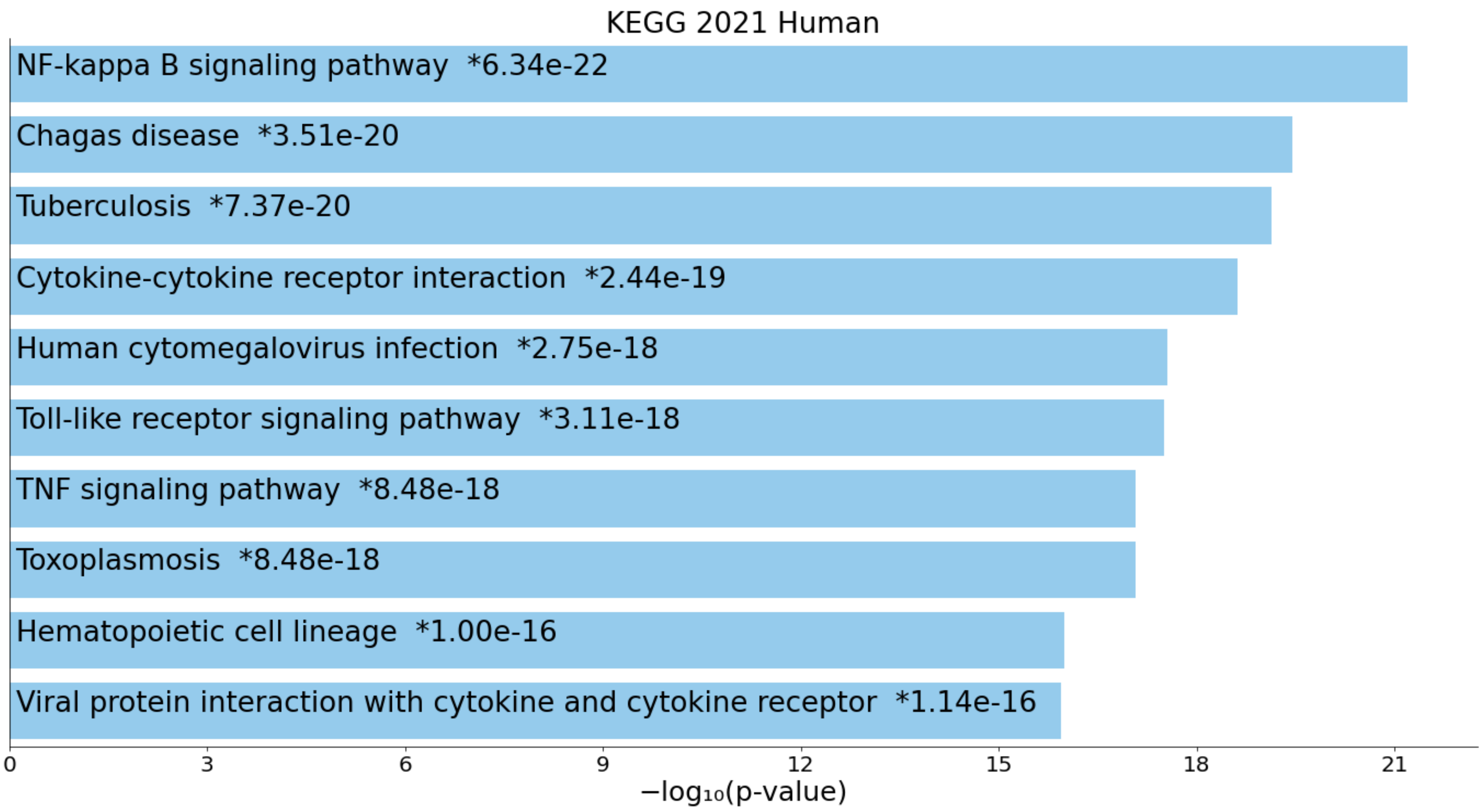

Figure 6. Heatmap (top-10) of the KEGG pathways accumulated in the differently expressed proteins of the expanded immune subnetwork of chronic fatigue spectrum disorders

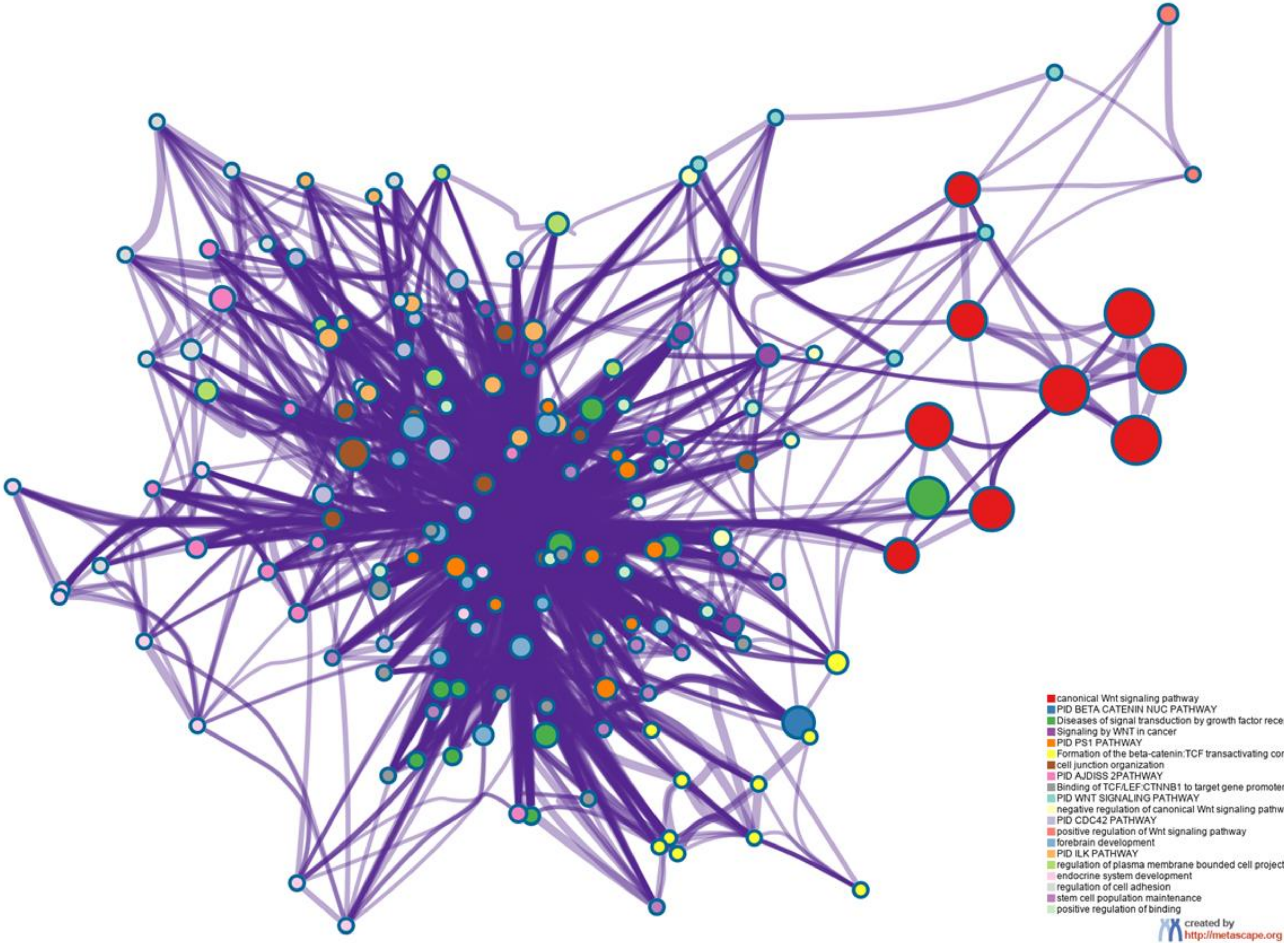
canonical Wnt signaling pathway
PID BETA CATENIN NUC PATHWAY
Diseases of signal transduction by growth factor rece
Signaling by WNT in cancer
PID PS1 PATHWAY
Formation of the beta-catenin:TCF transactivating cor
cell junction organization
PID AJDISS 2PATHWAY
Binding of TCF/LEF:CTNNB1 to target gene promoter
PID WNT SIGNALING PATHWAY
negative regulation of canonical Wnt signaling pathw
PID CDC42 PATHWAY
positive regulation of Wnt signaling pathway
forebrain development
PID ILK PATHWAY
regulation of plasma membrane bounded cell project
endocrine system development
regulation of cell adhesion
stem cell population maintenance
positive regulation of binding
created by
http://metascape.org

ESF 2, Figure 7. Enriched ontology term clusters in the Wnt/catenin subnetwork of chronic fatigue spectrum disorders. Terms are represented by a circle node, with the colors representing cluster identity and their size reflecting the number of input genes. One term is used to describe the clusters (colored labels). The thickness of the (bundled) edges linking the terms reflects the similarity score (threshold is > 0.3). The network is constructed and visualized using MetaScape and Cytoscape (v3.1.2).

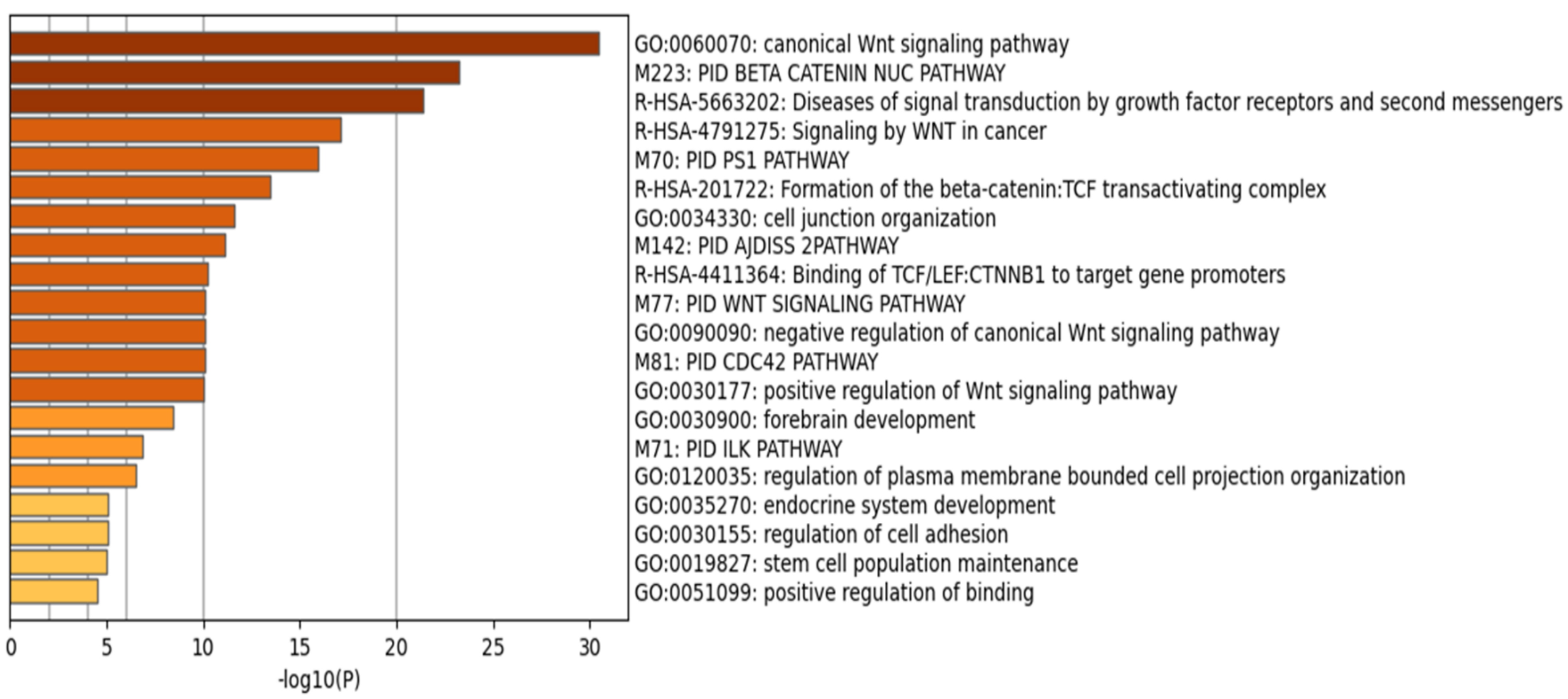

ESF 2, Figure 8. Heatmap of enriched ontology clusters showing the top-20 functions that were overexpressed in the Wnt/catenin subnetwork of patients with chronic fatigue spectrum disorders (accumulative hypergeometric p-values)

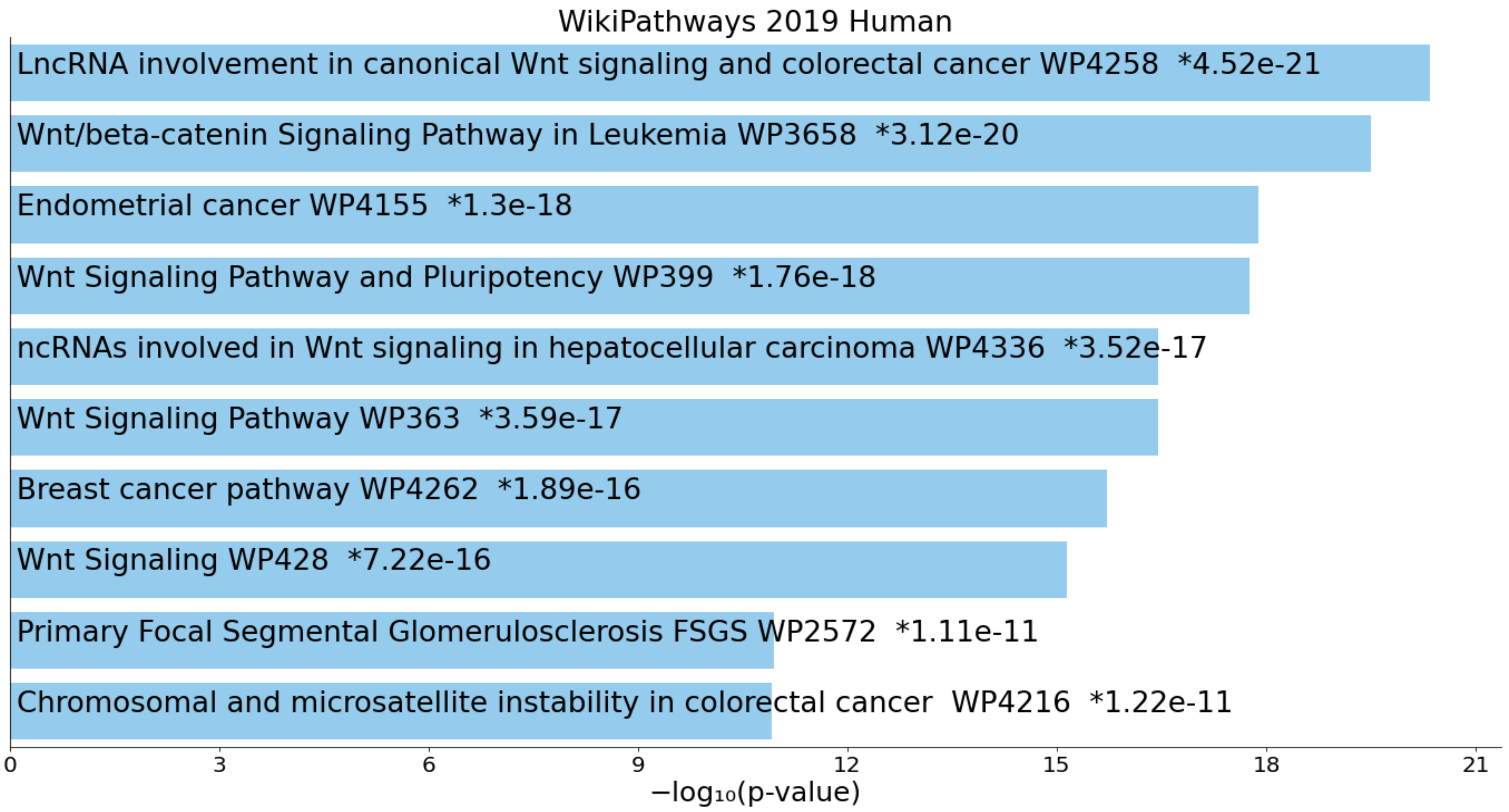

ESF 2, Figure 9. Heatmap (top-10) of the WikiPathways accumulated in the differently expressed proteins of the expanded Wnt subnetwork of chronic fatigue spectrum disorders

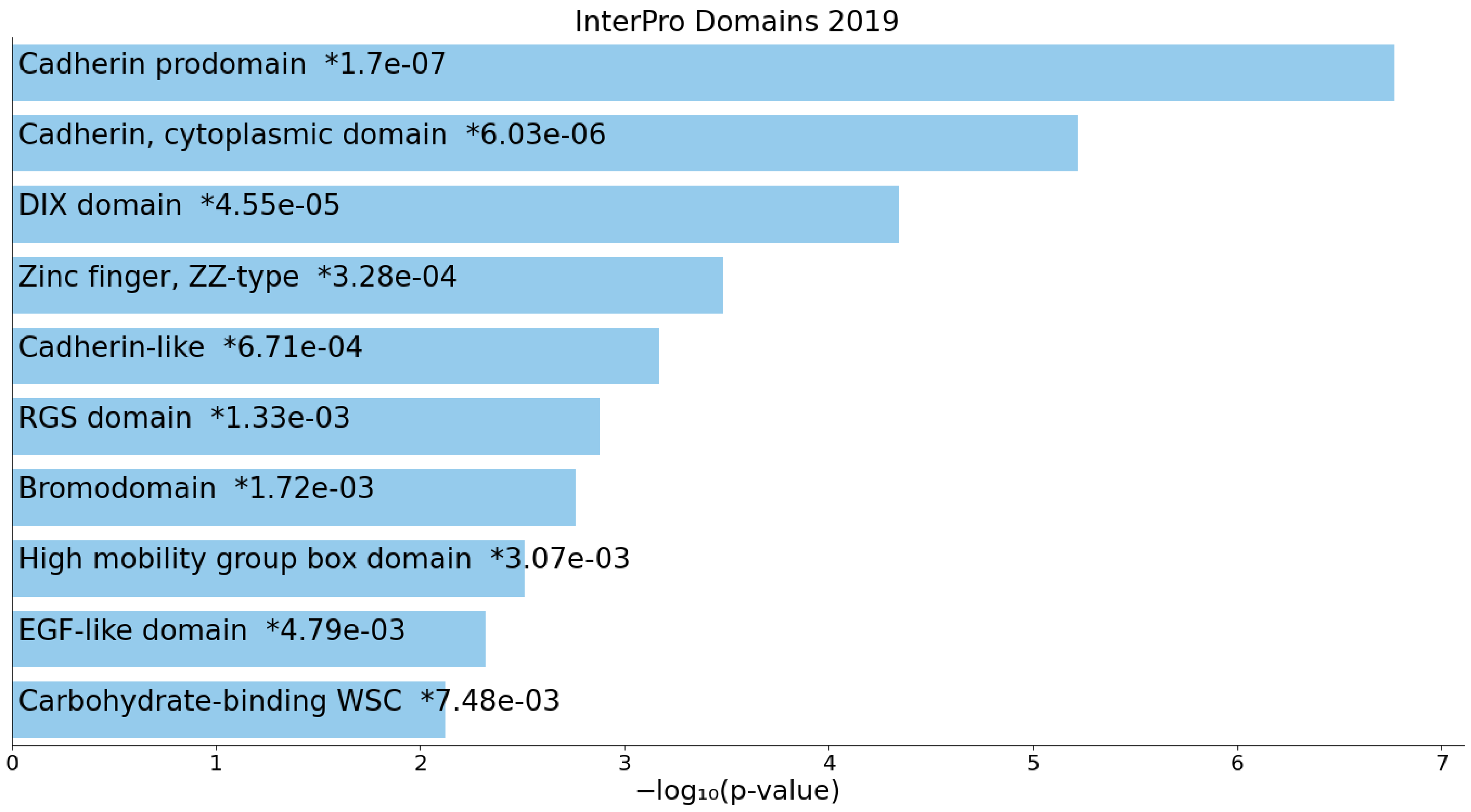

ESF 2, Figure 10. Heatmap (top-10) of enriched InterPro domain terms accumulated in the differently expressed proteins of the Wnt/catenin subnetwork of chronic fatigue spectrum disorders

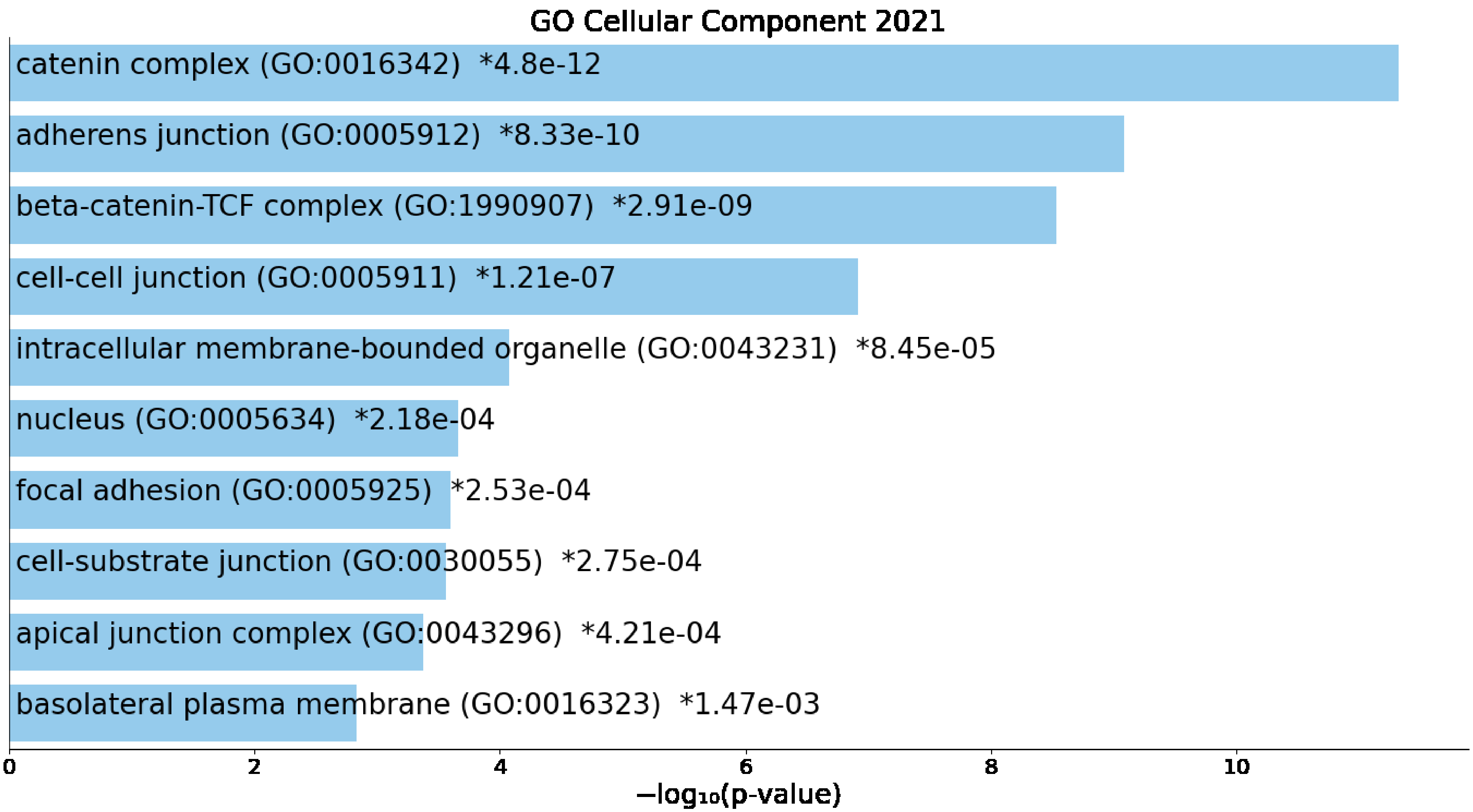

ESF 2, Figure 11. Heatmap (top-10) of enriched cellular component terms accumulated in the differently expressed proteins of the Wnt/catenin subnetwork of chronic fatigue spectrum disorders

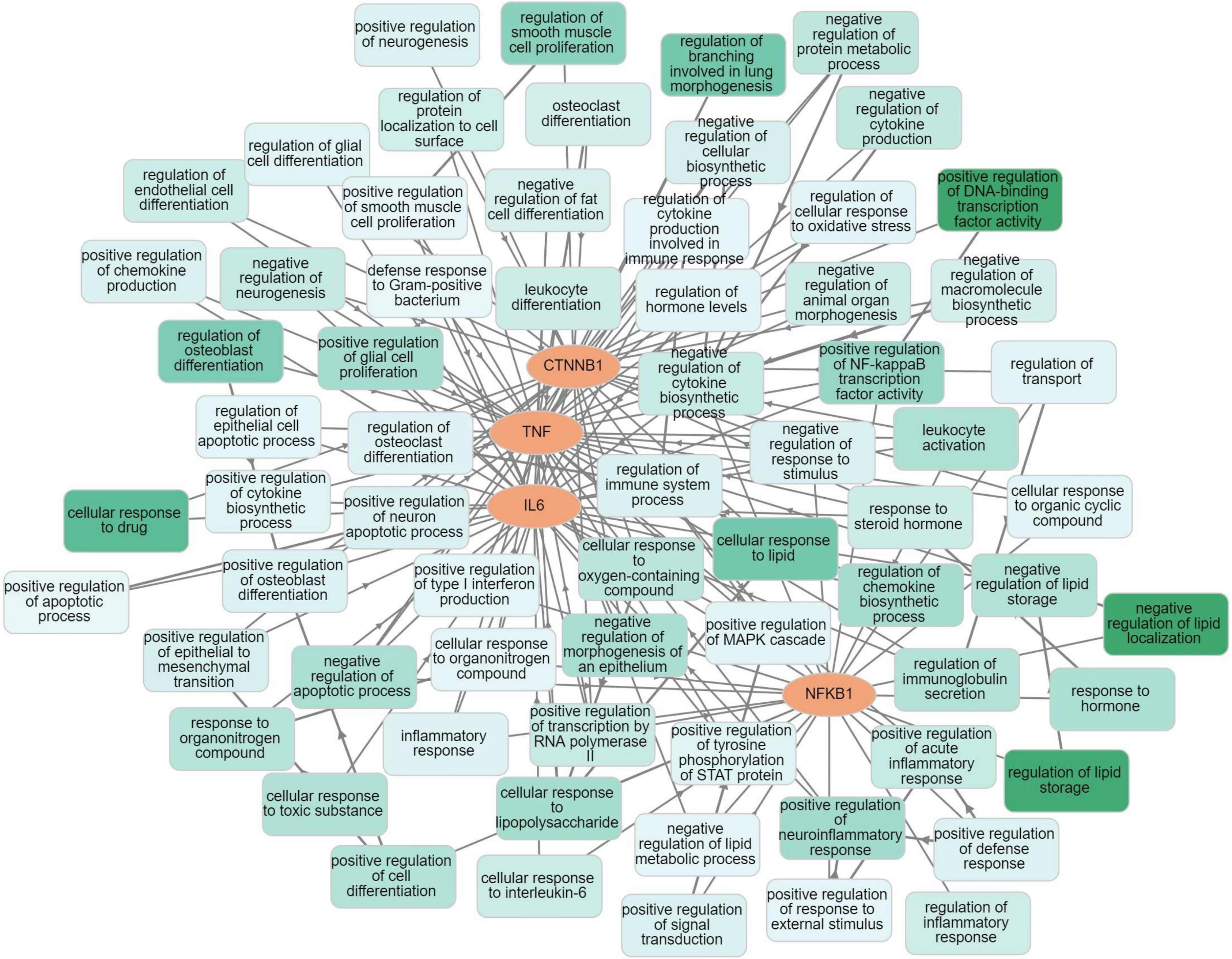

positive regulation of neurogenesis
regulation of smooth muscle cell proliferation
regulation of branching involved in lung morphogenesis
negative regulation of protein metabolic process
regulation of protein localization to cell surface
osteoclast differentiation
negative regulation of cellular biosynthetic process
negative regulation of cytokine production
regulation of glial cell differentiation
regulation of endothelial cell differentiation
positive regulation of smooth muscle cell proliferation
negative regulation of fat cell differentiation
regulation of cytokine production involved in immune response
regulation of cellular response to oxidative stress
positive regulation of DNA-binding transcription factor activity
positive regulation of chemokine production
negative regulation of neurogenesis
defense response to Gram-positive bacterium
leukocyte differentiation
regulation of hormone levels
negative regulation of animal organ morphogenesis
negative regulation of macromolecule biosynthetic process
regulation of osteoblast differentiation
positive regulation of glial cell proliferation
CTNNB1
negative regulation of cytokine biosynthetic process
positive regulation of NF-kappaB transcription factor activity
regulation of transport
regulation of epithelial cell apoptotic process
regulation of osteoclast differentiation
TNF
negative regulation of response to stimulus
leukocyte activation
regulation of immune system process
positive regulation of cytokine biosynthetic process
cellular response to drug
positive regulation of neuron apoptotic process
IL6
response to steroid hormone
cellular response to organic cyclic compound
cellular response to oxygen-containing compound
cellular response to lipid
positive regulation of osteoblast differentiation
positive regulation of type I interferon production
regulation of chemokine biosynthetic process
negative regulation of lipid storage
positive regulation of apoptotic process
negative regulation of lipid localization
negative regulation of morphogenesis of an epithelium
positive regulation of MAPK cascade
positive regulation of epithelial to mesenchymal transition
negative regulation of apoptotic process
cellular response to organonitrogen compound
regulation of immunoglobulin secretion
NFKB1
response to hormone
response to organonitrogen compound
inflammatory response
positive regulation of transcription by RNA polymerase II
positive regulation of tyrosine phosphorylation of STAT protein
positive regulation of acute inflammatory response
regulation of lipid storage
cellular response to toxic substance
cellular response to lipopolysaccharide
negative regulation of lipid metabolic process
positive regulation of neuroinflammatory response
positive regulation of defense response
positive regulation of cell differentiation
cellular response to interleukin-6
positive regulation of signal transduction
positive regulation of response to external stimulus
regulation of inflammatory response

ESF 2, Figure 12. Results of GOnet annotation visualization in chronic fatigue spectrum disorders depicting the hierarchical structure of GO terms and the hubs and master regulatory transcription factor

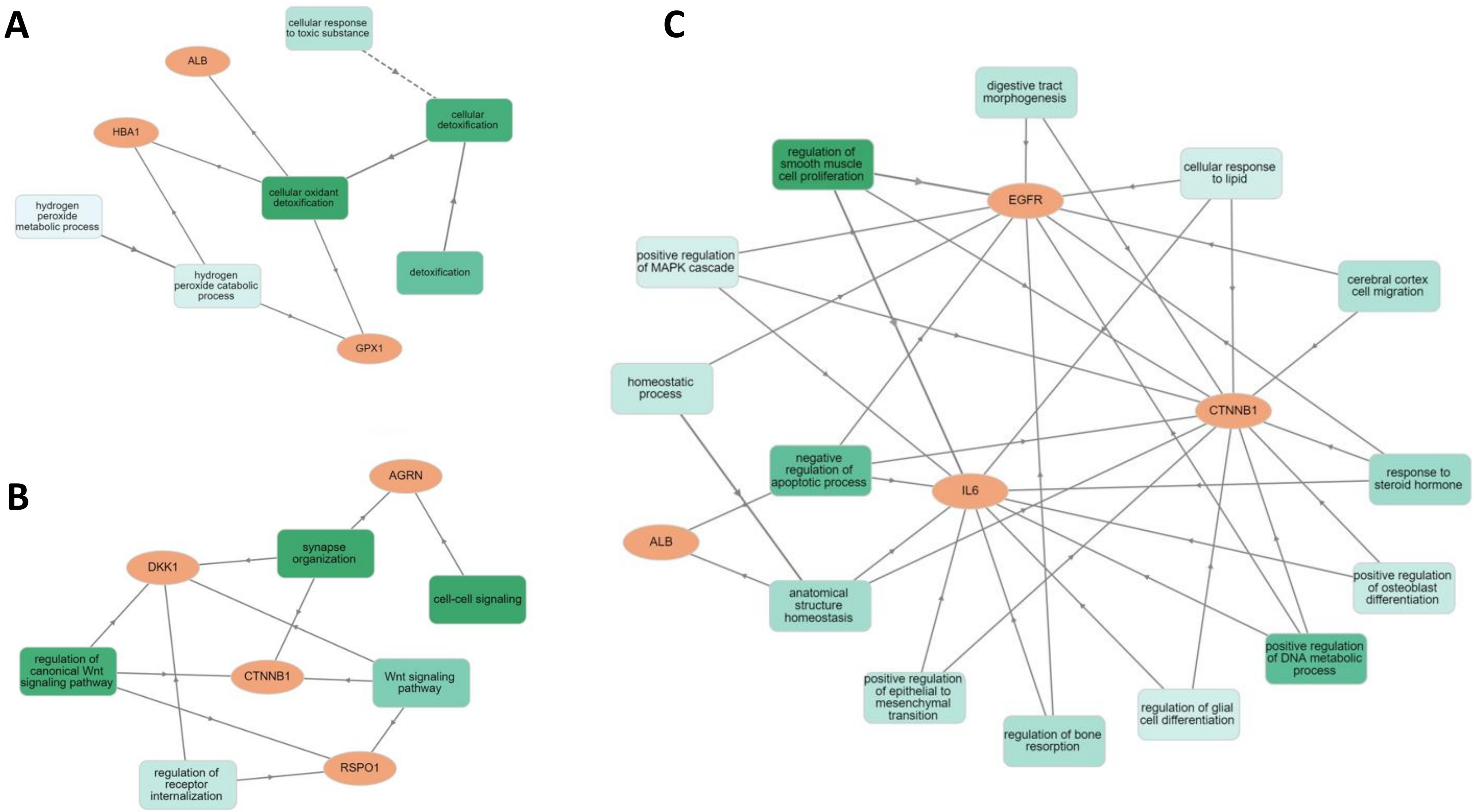
A
cellular response to toxic substance
ALB
HBA1
cellular detoxification
cellular oxidant detoxification
hydrogen peroxide metabolic process
hydrogen peroxide catabolic process
detoxification
GPX1
B
AGRN
synapse organization
DKK1
cell-cell signaling
regulation of canonical Wnt signaling pathway
CTNNB1
Wnt signaling pathway
RSPO1
regulation of receptor internalization
C
digestive tract morphogenesis
regulation of smooth muscle cell proliferation
EGFR
cellular response to lipid
positive regulation of MAPK cascade
cerebral cortex cell migration
homeostatic process
CTNNB1
negative regulation of apoptotic process
IL6
response to steroid hormone
ALB
anatomical structure homeostasis
positive regulation of osteoblast differentiation
positive regulation of DNA metabolic process
positive regulation of epithelial to mesenchymal transition
regulation of bone resorption
regulation of glial cell differentiation

ESF 2, Figure 13. Results of GOnet annotation visualization in chronic fatigue spectrum disorders depicting the hierarchical structure of GO terms and A: downregulated seed genes; B: seed genes of the Wnt/catenin pathways; and C: the hotspots in STRING enlarged networks

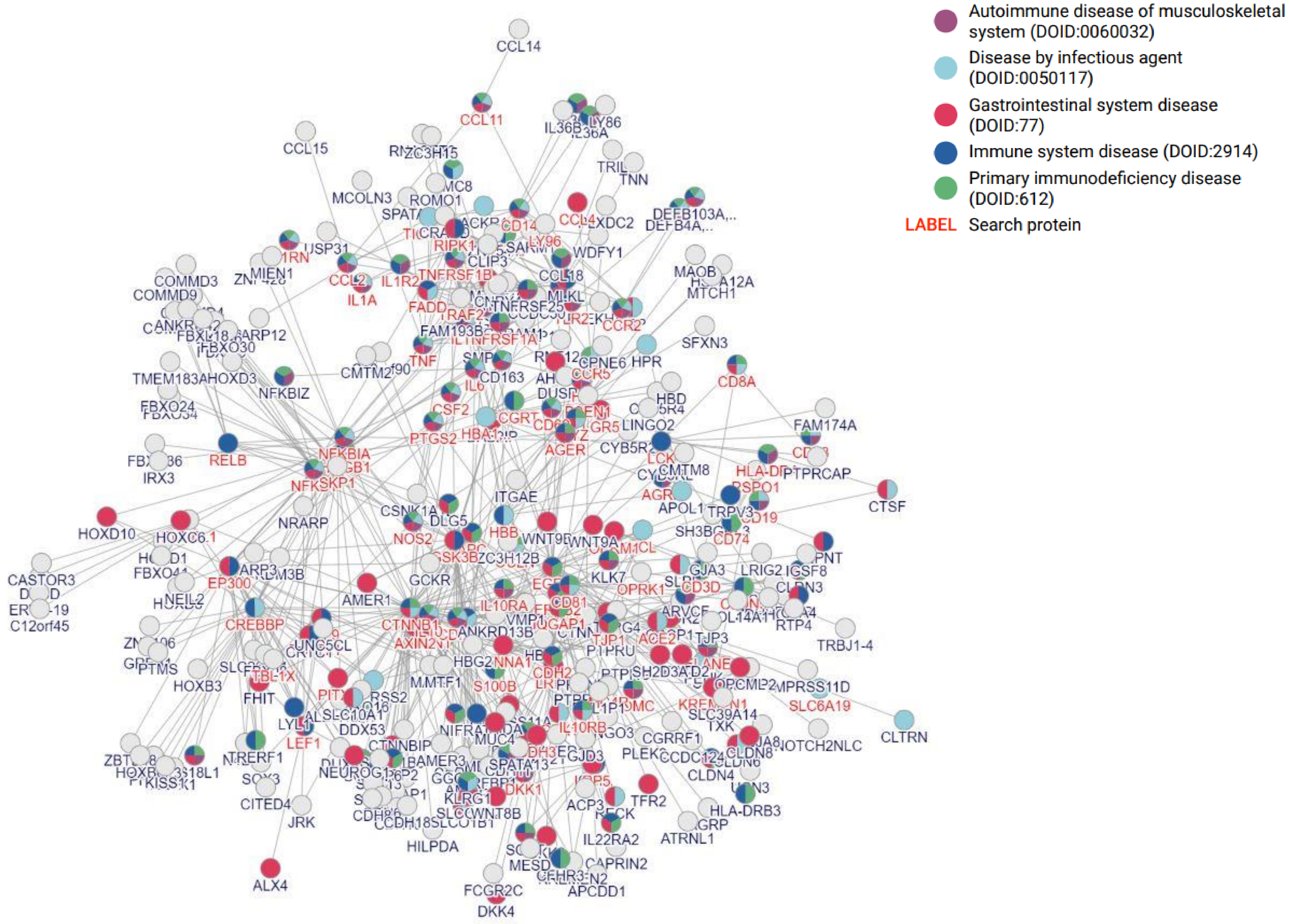
Autoimmune disease of musculoskeletal system (DOID:0060032)
Disease by infectious agent (DOID:0050117)
Gastrointestinal system disease (DOID:77)
Immune system disease (DOID:2914)
Primary immunodeficiency disease (DOID:612)
LABEL Search protein

ESF 2, Figure 14. An extended network constructed with inBio Discover showing the top Disease Ontology (DOID) annotations of chronic fatigue spectrum disorders
